# Synthesis and Assessment of Novel Probes for Imaging Tau Pathology in Transgenic Mouse and Rat Models

**DOI:** 10.1021/acschemneuro.0c00790

**Published:** 2021-03-10

**Authors:** Lindsay McMurray, Jennifer A. Macdonald, Nisha Kuzhuppilly Ramakrishnan, Yanyan Zhao, David W. Williamson, Ole Tietz, Xiaoyun Zhou, Steven Kealey, Steven G. Fagan, Tomáš Smolek, Veronika Cubinkova, Norbert Žilka, Maria Grazia Spillantini, Aviva M. Tolkovsky, Michel Goedert, Franklin I. Aigbirhio

**Affiliations:** † Molecular Imaging Chemistry Laboratory, Wolfson Brain Imaging Centre, University of Cambridge, Cambridge CB2 0QQ, United Kingdom; ‡ MRC Laboratory of Molecular Biology, Cambridge CB2 0QH, United Kingdom; § Department of Clinical Neurosciences, University of Cambridge, Cambridge CB2 0QQ, United Kingdom; ∥ Axon Neuroscience R&D Services SE, Bratislava, Slovak Republic 811 02

**Keywords:** PET, imaging, tau, neurodegeneration, mouse, rat

## Abstract

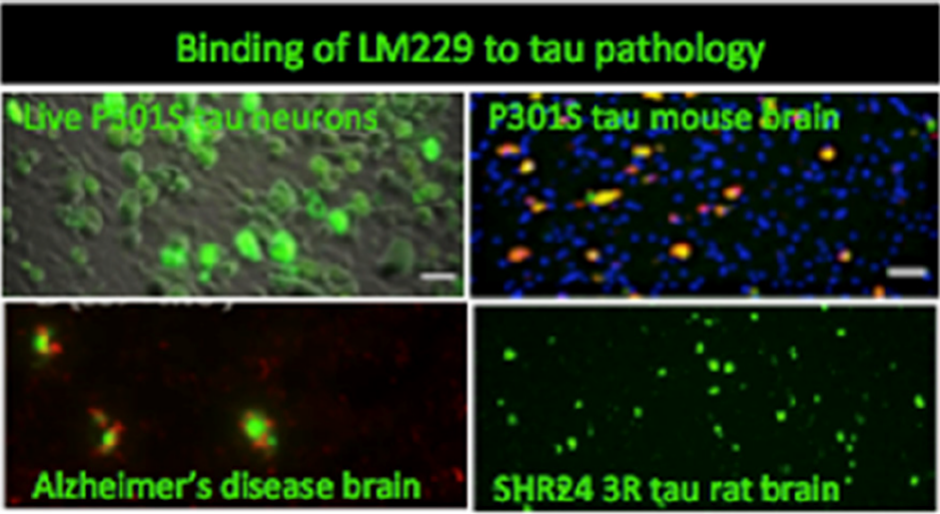

Aggregated tau protein is a core pathology present in several neurodegenerative diseases. Therefore, the development and application of positron emission tomography (PET) imaging radiotracers that selectively bind to aggregated tau in fibril form is of importance in furthering the understanding of these disorders. While radiotracers used in human PET studies offer invaluable insight, radiotracers that are also capable of visualizing tau fibrils in animal models are important tools for translational research into these diseases. Herein, we report the synthesis and characterization of a novel library of compounds based on the phenyl/pyridinylbutadienylbenzothiazoles/benzothiazolium (PBB3) backbone developed for this application. From this library, we selected the compound LM229, which binds to recombinant tau fibrils with high affinity (*K*_d_ = 3.6 nM) and detects with high specificity (a) pathological 4R tau aggregates in living cultured neurons and mouse brain sections from transgenic human P301S tau mice, (b) truncated human 151-351 3R (SHR24) and 4R (SHR72) tau aggregates in transgenic rat brain sections, and (c) tau neurofibrillary tangles in brain sections from Alzheimer’s disease (3R/4R tau) and progressive supranuclear palsy (4R tau). With LM229 also shown to cross the blood–brain barrier *in vivo* and its effective radiolabeling with the radioisotope carbon-11, we have established a novel platform for PET translational studies using rodent transgenic tau models.

## Introduction

Imaging protein aggregates, characteristic features of a variety of neurodegenerative diseases,^1^ has become a powerful means of investigating their pathology. In particular, positron emission tomography (PET) is the method of choice to study their pathophysiology *in vivo* using imaging probes selective for the various aggregated protein forms. PET probes were initially developed for imaging β-amyloid (Aβ) aggregated protein, present as plaque deposits in Alzheimer’s disease (AD),^2^ then probes selective for protein tau aggregates, which form intracellular neurofibrillary tangles (NFT) in AD.^3,4^ In addition to AD, pathological tau aggregates are a constituent feature of several other neurodegenerative disorders, collectively termed tauopathies;^5−7^ hence tau PET imaging is a key biomarker technology. Since the first reports of [^18^F]T807 ([^18^F]AV1451)^8^ as a suitable PET radiotracer for selective imaging of NFTs, several radiotracers capable of binding to and visualizing tau have now been used for clinical PET studies,^4,9^ including [^11^C]PBB3,^10^ [^18^F]PI-2620,^11^ and [^18^F]MK6240.^12^

While the final aim is to employ tau PET probes for human diagnostics and investigation of disease mechanisms, there is a need to underpin their application by studying experimental animal models of neurodegeneration. These provide a critical translational link between laboratory research from cellular and animal models to human disease and, conversely, allow human pathophysiological data to be used to design more appropriate experimental models and studies. In particular, transgenic animal models of dementia are important for understanding the involvement of Aβ and NFTs in disease, especially given their use in longitudinal and behavioral studies.^13^ Therefore, the development of PET radiotracers that are capable of visualizing tau aggregates within these animal models is of great importance in advancing both the understanding of the disease and the development of new therapeutics alongside clinical studies.

While the present range of tau PET radiotracers has shown excellent affinities for tau fibrils, they are still limited in their ability to visualize tau NFTs in animal models as well as in humans. For example, [^18^F]T807 (AV1451) showed no specific binding to tau tangles in transgenic mice expressing mutant human P301L tau as assessed by *in vitro*(14) and microPET *in vivo*(15) imaging studies. Studies with P301S tau mice indicated a difference in retention of [^18^F]AV1451 in their brainstem of no more than 23% compared to control wild-type (WT) mice.^16^ More successful have been the radiotracers [^11^C]PBB3, which has been shown to bind to tau pathology in PS19 tau transgenic mice^10^ and [^18^F]THK5117, which bound to tau aggregates in two transgenic mice mouse models, P301S tau and GSK3-β X P301L tau (biGT).^17^ Even though these can detect tau fibrils, higher specificity is needed to analyze subtle early changes in tau aggregation. Notably, imaging tau pathology in a rat model of tauopathy has yet to be reported. With the rat brain being approximately six times larger than the mouse brain, this would bring distinct advantages for research involving small animal PET imaging. For example, it would enable more accurate quantification of radioactivity concentration in brain regions and blood sampling for gold standard blood-based kinetic analysis.

**Scheme 1:**
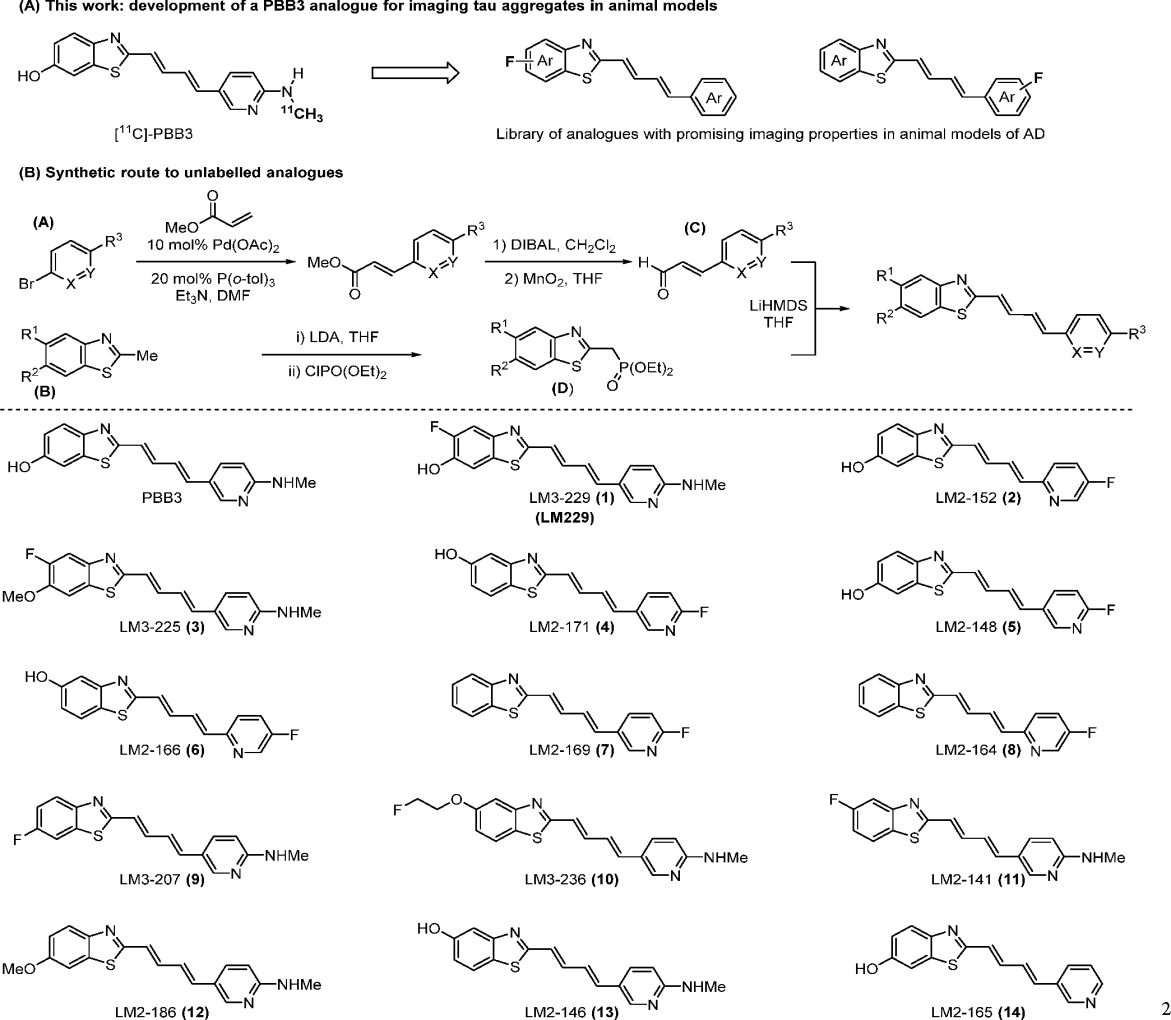
(A) Development of Novel Compounds for Imaging Tau Aggregation in Animal Models of Tau and (B) Streamlined Convergent Synthetic Route for a Compound Library

Therefore, to improve the development of PET imaging probes for visualizing tau pathology in transgenic models of neurodegeneration, herein, we report the synthesis and characterization of a novel series of compounds based on the phenyl/pyridinylbutadienylbenzothiazoles/benzothiazolium (PBB3) structure (Scheme 1A), which has been shown to bind to tau aggregates in both mouse models and humans.^10^ Binding affinities to heparin-induced synthetic recombinant tau fibrils were determined as well as binding to aggregated tau in a transgenic human P301S tau mouse model.^18^ The most promising compound from this series, LM229, was then assessed for its binding to pathological tau in transgenic mouse and rat brain tissues *in vitro* and *in vivo* and in two human tauopathies. The successful radiolabeling of LM229 with the carbon-11 (*t*_1/2_ = 20 min) PET radioisotope is described.

## Results and Discussion

### Synthesis of a Compound Library

At the outset of our investigation, a library of unlabeled analogues based on the core structure of PBB3^19^ was synthesized. To rapidly access compounds of interest, we developed a streamlined convergent route centered on a Horner–Wadsworth–Emmons coupling of two key fragments: aldehyde **C** and phosphonate **D** (Scheme 1B). Synthesis of aldehyde **C** commenced with palladium(II)-catalyzed Heck reaction of a range of commercially available bromopyridines (**A**) with methyl acrylate. DIBAL reduction followed by immediate benzylic oxidation with manganese dioxide furnished the desired aldehyde **C**. The desired benzothiazoles (**B**) were either available from commercial sources or synthesized from simple starting materials. Deprotonation with lithium diisopropylamide (LDA) followed by addition of diethylchlorophosphate afforded the desired phosphonates **D**. Coupling of the aldehyde **C** and phosphonate **D** proceeded smoothly for all derivatives to furnish the desired library of PBB3 analogues.

### Assessment of Binding Affinities

All compounds were assessed for their ability to bind to synthetic recombinant tau fibrils using a fluorescence quenching assay^20^ (Table 1), for which we first established a *K*_d_ value for PBB3 of 1.4 nM, in good agreement with the previously reported value of 1.3 nM.^21^ Binding affinities for some compounds (141, 164, 166, 169, 171, and 207) could not be assessed with this assay because of negligible changes in fluorescence intensity. While several compounds showed affinities below the micromolar range, LM3-229 (abbreviated LM229 or 229) showed a *K*_d_ of 3.8 nM, in the nanomolar range expected of a PET ligand, combined with having chemical moieties with the potential to be radiolabeled by either carbon-11 or fluorine-18 for development as a PET imaging probe. This compound was then selected for further characterization and development.

**Table 1:** *K*_d_ (nM) of Compound Binding to Heparin-Induced Synthetic Tau Fibrils Determined by Fluorimetry

name	R1	R2	R3	X	Y	*K*_d_ (nM)
PBB3	H	OH	NHMe	C(H)	N	1.4
148	H	OH	F	C(H)	N	56
152	H	OH	F	N	C(H)	353
229	F	OH	NHMe	C(H)	N	3.6
225	F	OMe	NHMe	C(H)	N	1.1
171^a^	OH	H	F	C(H)	N	
166^a^	OH	H	F	N	C(H)	
169^a^	H	H	F	C(H)	N	
164^a^	H	H	F	N	C(H)	
236	OCH_2_CH_2_F	H	NHMe	C(H)	N	454
207^a^	H	F	NHMe	C(H)	N	
141^a^	F	H	NHMe	C(H)	N	

a Due to negligible change of fluorescence intensity, binding affinities could not be determined.

### Binding to Transgenic P301S Tau Mouse Brain Sections

**Figure 1:**
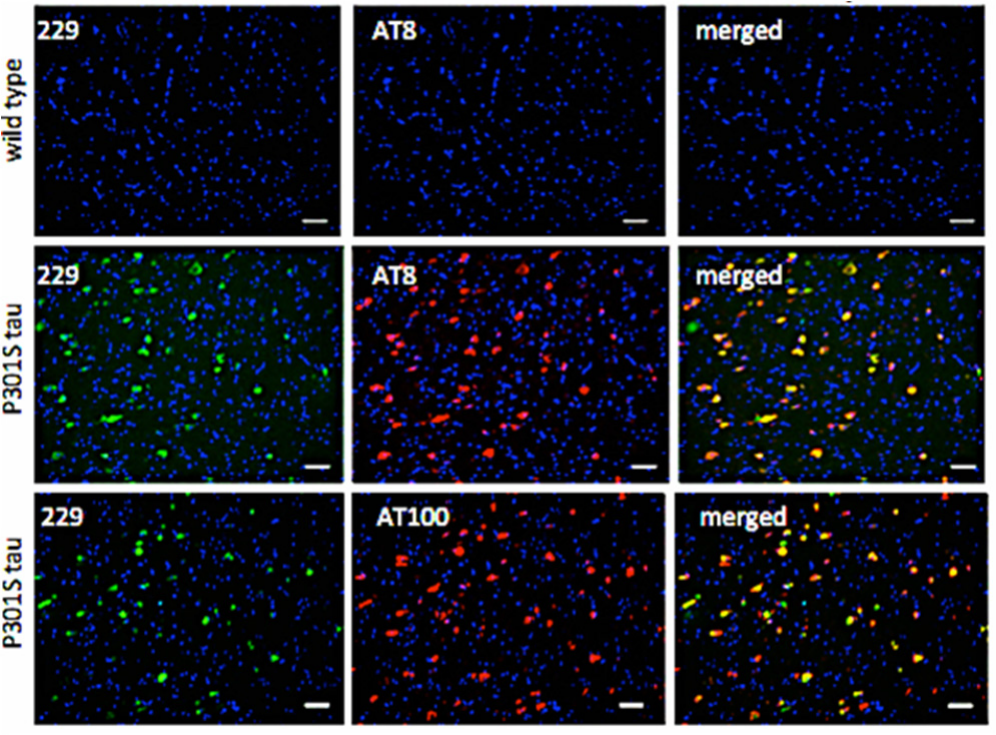
LM229 binding to mouse brain sections is specific to neurons with pathological tau aggregates. (Top) No specific binding to brainstem sections from WT animals but coincident LM229 binding (green) and (middle) AT8 or (bottom) AT100 antibody staining (red) in phenotypic P301S tau mice; AT100 binding correlates with tau fibrils. Scale bar = 20 μm.

We then screened all of the compounds for their binding to tau inclusions in brain sections from transgenic P301S tau mice, which express the shortest isoform (0N4R) of human 4-repeat (4R) tau,^18^ using their autofluorescence properties to rapidly analyze their binding. Although some of the compounds recognized tau aggregates, they also showed significant fluorescence in WT brain sections, giving a high background and low specificity, therefore unlikely to be useful as PET radiotracers. However, compound LM229, showed intense, specific binding that correlated with hyperphosphorylated pathological tau (AT8 antibody, pS202/pS205) and β-sheet-rich filamentous tau (AT100 antibody, pT212/pS214/pT217^18,22^), consistent with its high binding affinity to recombinant tau fibrils (Figure 1).

To further confirm that binding of LM229 was specific to aggregated tau rather than to soluble tau, binding to brain sections from P301S tau mice at 2 and 6 months of age was compared, based on the fact that a significant amount of tau aggregates is detected in older mice (>3 months).^18,23^ Rare binding was detected in brain sections from 2 months old mice, but significant binding to large populations of neurons was evident at 6 months of age (Supporting Information, Figure S1).

**Figure 2:**
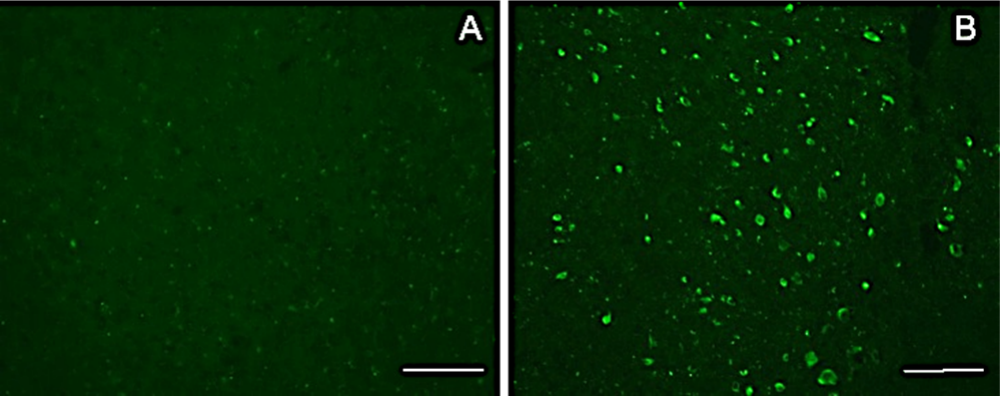
Fluorescence image of brain section from WT (A) and P301S tau mice (B) fixed 1 h after i.v. injection of LM229 showing entry into brain from periphery. Scale bar = 100 μm.

**Figure 3:**
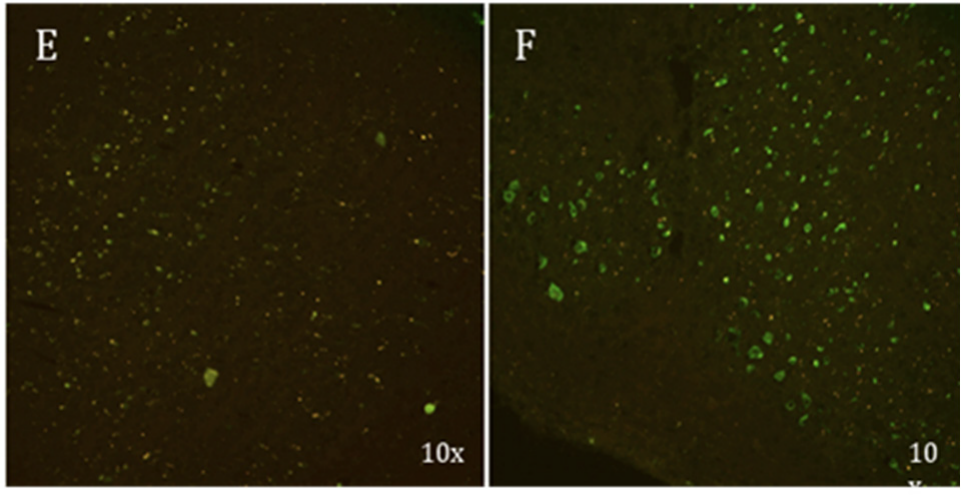
P301S tau brain tissue from (E) *ex vivo* experiments with PBB3 (i.v. injection 1 h prior to injection) colocalized with AT8 showing binding of PBB3 with significant nonspecific binding; (F) *ex vivo* experiments with LM229 showing significant specific binding to tau aggregates as confirmed with colocalization with AT8.

We next looked at the ability of LM229 to cross the blood–brain barrier (BBB), a key property of any viable neuro-radiotracer. LM229 was injected intravenously (i.v.) (1 mg/kg weight), and after 1 h, WT and P301S tau mice were perfused-fixed and brain sections were imaged under a fluorescence microscope. Figure 2 shows binding of LM229 to some nerve cells from P301S tau transgenic mice, with little binding to the brains of non-transgenic control mice., confirming that the compound crosses the BBB. Moreover, when compared with PBB3 (Figure 3), and based on the compounds having similar extinction coefficients, LM229 showed comparably less non-specific binding and more specific binding to tau aggregates by fluorescence microscopy, suggesting that LM229 was highly suitable as a ligand for tau aggregate tracing within the transgenic mouse model. However, we await a comparison of PBB3 and LM229 by PET imaging to determine their relativity properties as *in vivo* imaging probes.

### Binding of LM229 to Cultured Neurons from P301S Tau Transgenic Mice

**Figure 4:**
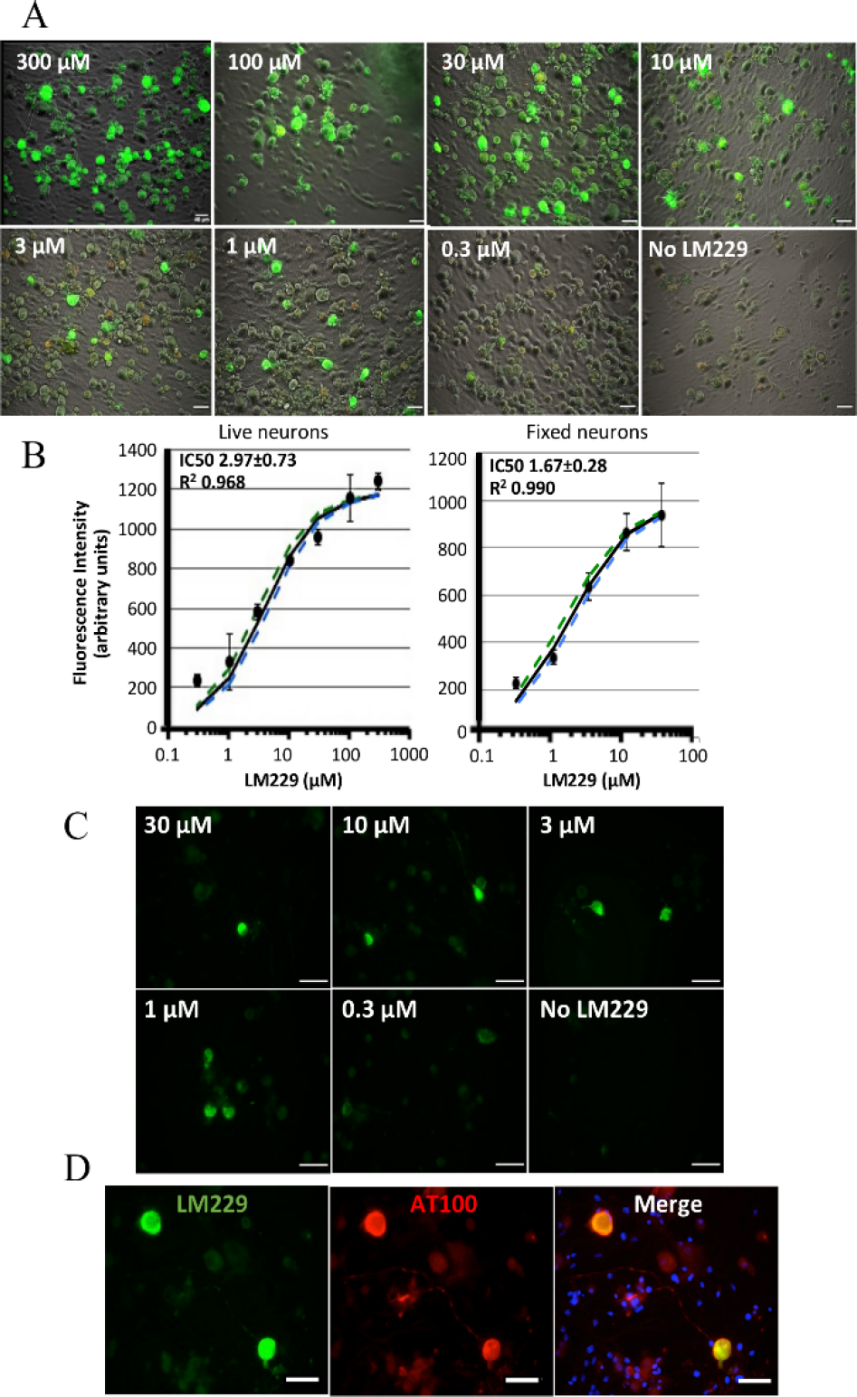
(A) Fluorescence images of LM229 binding to live DRG neurons (green). Phase contrast images show total live neurons. (B) Fluorescence intensity per neuron as a function of LM229 concentration (mean ± SD, 5–15 neurons per concentration, three independent cultures; black line, result of nonlinear curve fitting, green and blue dashed lines, 95% confidence intervals; live neurons: IC_50_ = 2.97 ± 0.73; *R*^2^ = 0.968; fixed neurons: IC_50_ = 1.67 ± 0.28; R^2^ = 0.990). (C) Dose–response of LM229 binding to fixed neurons. (D) Co-staining of LM229 (10 μM, green) and antiphospho-tau antibody AT100 (red). Blue in merged image shows cell nuclei in the culture.

P301S tau mice with aggregated tau in the brain also express aggregated tau in DRG neurons.^24−26^ Unlike neurons from the brain, which do not survive in culture when extracted beyond neonatal ages, DRG neurons with tau filaments can be cultured from adult mice when filamentous tau pathology is well-developed. To estimate the apparent affinity of LM229 to tau aggregates directly in living neurons, we imaged live neurons after 20 min incubation with various concentrations of LM229 (Figure 4A). Analysis of fluorescence intensity by nonlinear curve fitting to a Michaelis–Menten equation yielded an IC_50_ of 2.7 ± 0.7 μM (*R*^2^ = 0.97) (Figure 4B). A dose–response conducted after fixation and permeabilization of the neurons (Figure 4C) gave a similar affinity (IC_50_ = 1.7 ± 0.3 μM, *R*^2^ = 0.99, Figure 4B), showing that the plasma membrane does not form a barrier to LM229 entry/binding. LM229 binding was specific to neurons with aggregated tau, as detected with the AT100 antibody^18,22,23,26^ (Figure 4D). Notably, inspection of the neurons 3 days after LM229 exposure and washes showed that the LM229 fluorescence was still retained albeit to a lower intensity. Although PBB3 also bound to DRG neurons with tau aggregates at 30–100 μM, it was not possible to derive an IC_50_ value because of its low affinity in this assay (Supporting Information, Figure S2). Thus, LM229 is highly cell permeable, and binding follows a single saturable binding algorithm. The shift from nanomolar to micromolar affinity between binding to heparin tau fibrils and tau aggregates in P301S tau neurons may reflect the different environment and assay conditions or the different conformations that tau aggregates assume in neurons compared to heparin–tau complexes.^27^

### Binding to Tau Transgenic Rat Brain Tissue

**Figure 5:**
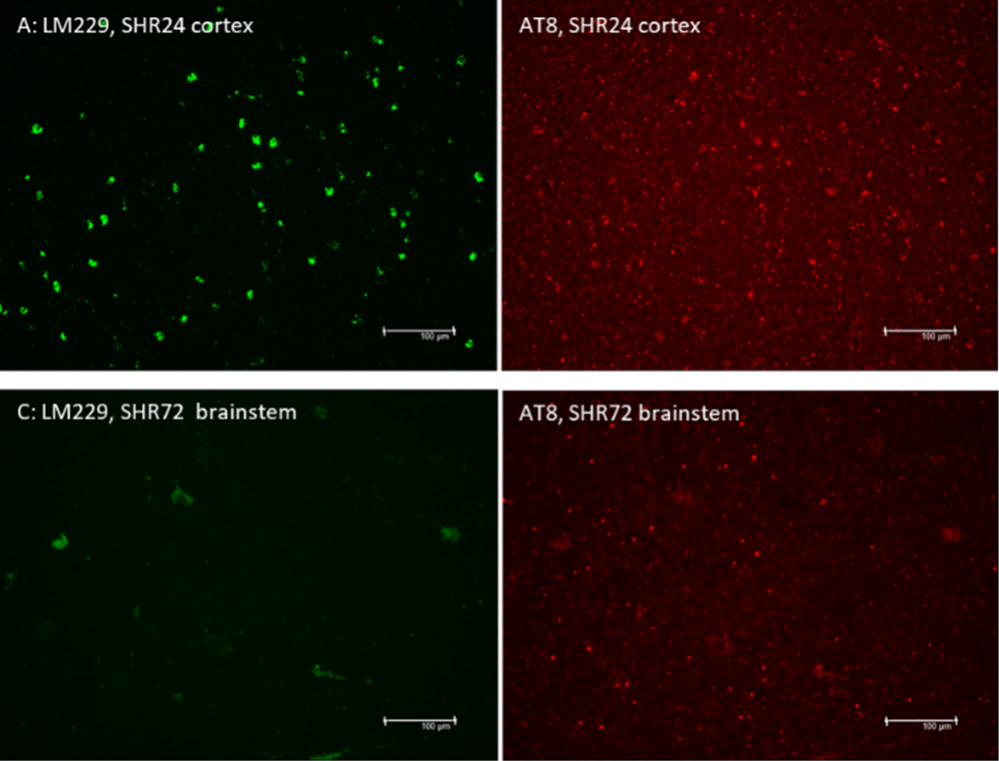
LM229 (green) binding to (A) 3R tau in cortical sections from SHR24 rat brains and (B) 4R tau in brainstem sections from SHR72 rats. (C, D) Colocalization with AT8 (red).

**Figure 6:**
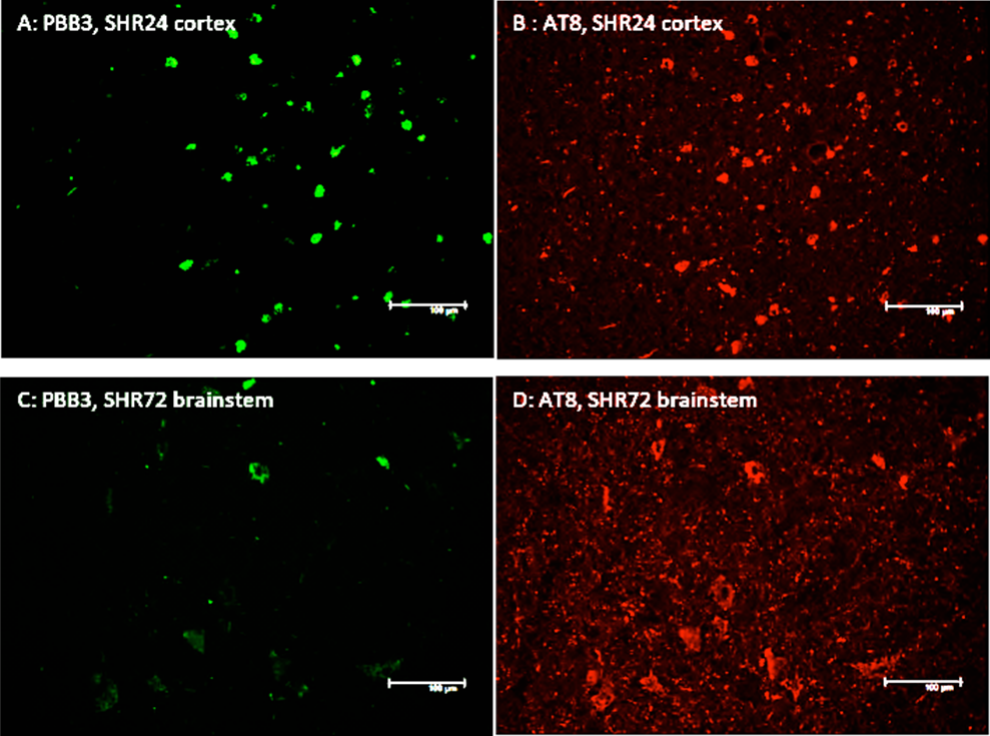
Binding of PBB3 (green) to (A) 3R tau in the cortex of SHR24 brain tissue and (B) to 4R tau in the brainstem of SHR72 and (C, D) colocalization with staining AT8 (red).

Since future PET studies for development of these compounds would require kinetic modeling for their assessment *in vivo*, we tested their performance in tau transgenic rats. Rat brains and blood volumes are about 6- and 10-fold larger, respectively, than those of mice and thus enable higher resolution imaging and ample volume for repeated blood sampling. We used two transgenic rat lines expressing human-truncated tau comprising the proline-rich region and either three microtubule binding domains (3R tau151-391, SH24)^28^ or four microtubule binding domains (4R tau151-391, SHR72).^29^ Both LM229 (Figure 5A) and PBB3 (Figure 6A) bound with similar properties to pathological aggregated tau in brain sections from SHR24 rats, which develop tangle pathology in the cortex, as confirmed by colocalization with AT8 immunofluorescence (Figures 5B and 6B). Likewise, with similar properties, both LM229 (Figure 5C) and PBB3 (Figure 6C) bound to tau in brain sections from SHR72 rats, which develop tangles mainly in the brainstem, as confirmed by colocalization with AT8 immunofluorescence (Figures 5D and Figure 6D). However, compared to the SHR24 rat brain sections, there were less inclusions detected, which seems to indicate the compounds have preferential binding toward the three repeat forms of tau in these rat models. To our knowledge, this is the first time that tau PET imaging compounds have been shown to bind to pathological tau in a rat transgenic model. These results establish the basis to perform *in vivo* PET imaging studies, including further investigations into the selectivity of LM229 toward the three and four repeat forms of tau.

### Binding to Human Tau Pathology

**Figure 7:**
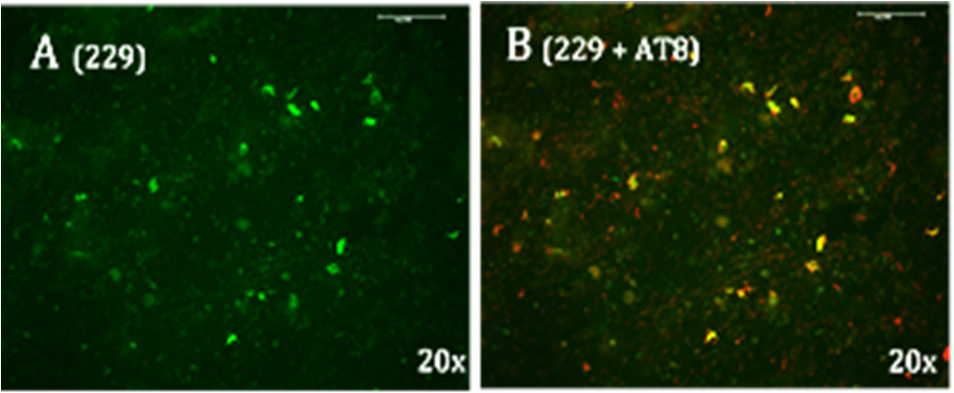
(A) *Post-mortem* PSP tissue stained with LM229; (B) Double staining of PSP tissue with LM229 and AT8 (red) to confirm colocalization of LM229 with tau aggregates.

**Figure 8:**
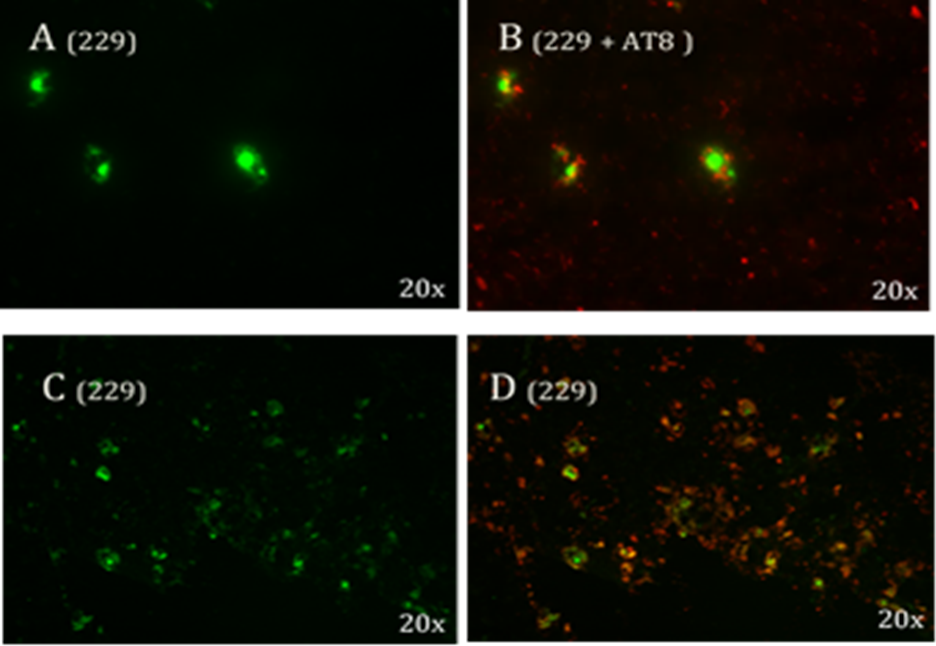
*Post mortem* AD tissue stained with LM229 and (A,C) and double staining with AT8 (B) and beta-amyloid antibodies (D), suggesting binding to tau rather than amyloid aggregates.

To further validate LM229 as a potential radiotracer for translational research, we investigated its binding to tau aggregates in human globus pallidus/putamen from progressive supranuclear palsy (PSP) subjects (Figure 7) and its selectivity for tau over β-amyloid pathology in AD frontal cortex sections (Figure 8). An intense signal was emitted by LM229 binding to PSP sections (Figure 6A), which was extensively colocalized with AT8 binding.

In slices from frontal cortex of AD brains, LM229 gave rise to strong signal (Figure 8A), which colocalized with AT8 immunofluorescence (Figure 8B). In contrast, LM229 fluorescence intensity that colocalized with Aβ (Figure 8C) was lower (Figure 8D),suggesting selectivity for tau *versus* β-amyloid protein.

### Radiosynthesis of [^11^C]LM229

**Scheme 2:**
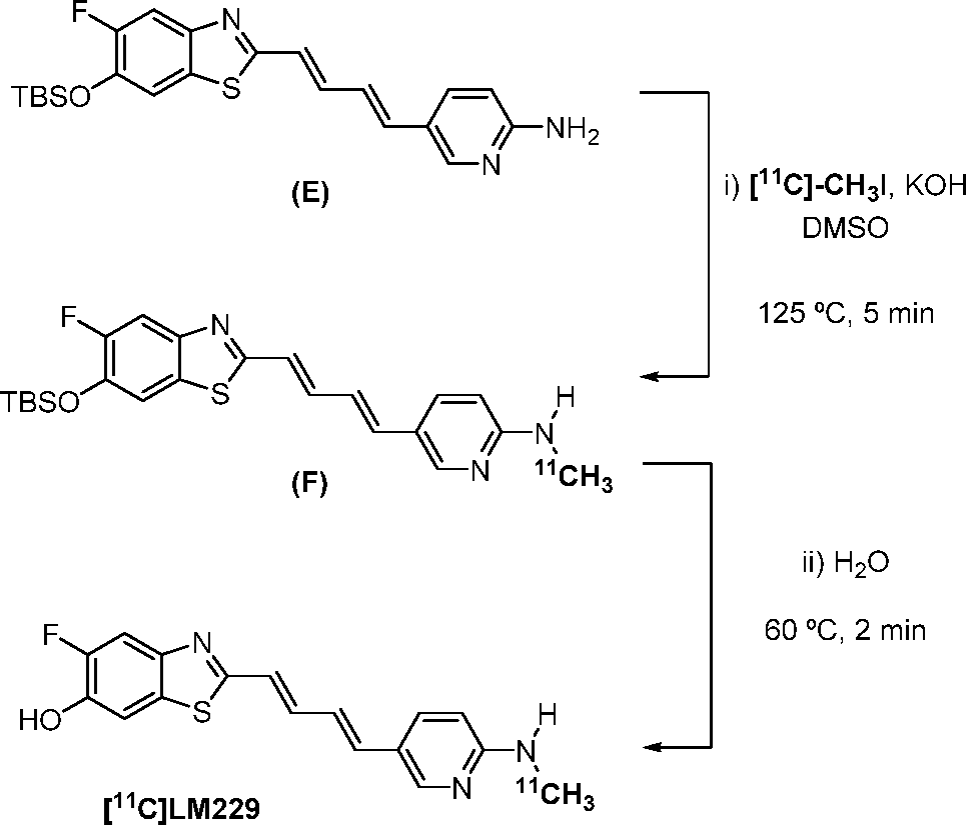
Two-Step Radiosynthesis of [^11^C]LM229

Having characterized the *in vitro* properties of unlabeled LM229, we next synthesized a [^11^C]radiolabeled version for application as a PET imaging probe. An analogous route to that reported by Wang *et al.*(19) for the synthesis of [^11^C]PBB3 was developed. [^11^C]methyl iodide was trapped in a mixture of *tert*-butyldimethylsilyl (TBS)-protected **E** and potassium hydroxide in dimethylsulfoxide. The resulting mixture was heated to 125 °C for 5 min to access intermediate **F**. Addition of water, followed by heating to 60 °C for 2 min, enabled the deprotection of the TBS group to furnish [^11^C]LM229 (Scheme 2). It was found that a loading of potassium hydroxide higher than that reported was required to prevent formation of undesired side products. Due to the heterogeneity of the reaction, mixing of the suspension of **E** and KOH *via* vortex prior to the reaction also proved to be crucial in the formation of [^11^C]LM229. Purification of the crude reaction mixture by semipreparative HPLC yielded [^11^C]LM229 (Supporting Information, Figure S1), which was reformulated into ethanolic saline using a C18-light Sep-Pak cartridge to afford [^11^C]LM229 1.0–1.4 GBq at end-of-synthesis (10% DCY, 96.6% RCP, 250 GBq/μmol molar activity).

### PET Studies with [^11^C]LM229

**Figure 9:**
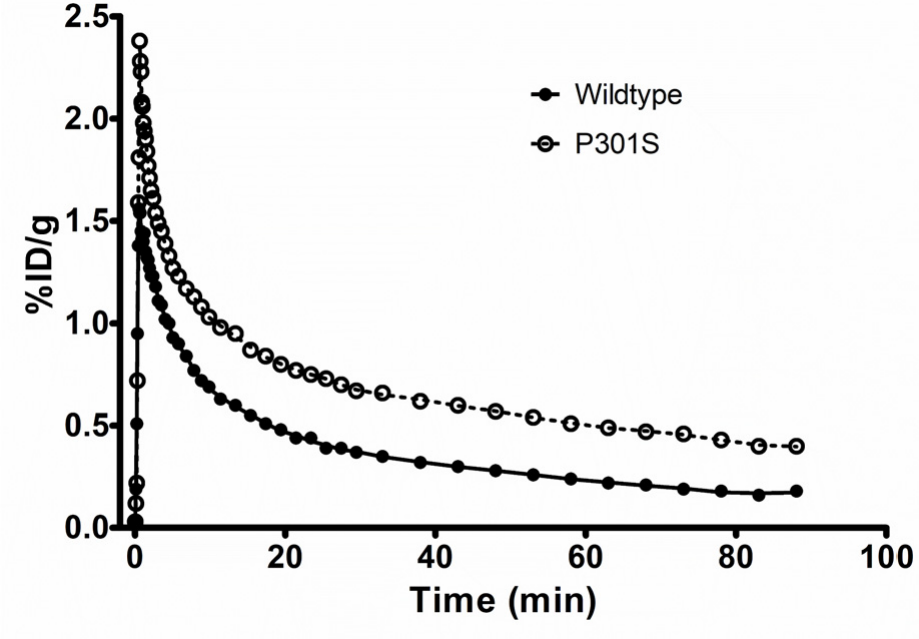
Time–activity curves for [^11^C]LM229 from *in vivo* PET studies in wild-type and P301S mice.

We then performed preliminary *in vivo* PET studies with [^11^C]L229 in both WT and transgenic P310S tau mice. These studies confirmed blood–barrier brain penetration by the radiotracer with maximum brain uptake (%ID/g max; WT = 1.56, P301S = 2.38) within the first minute followed by washout within the 90 min scan (Figure 9). The brain exposure was higher in the P301S mouse (area under the curve, AUC = 60) compared to the wild-type mouse (AUC = 34). Further PET studies are required to fully characterize the *in vivo* pharmacokinetics of the radiotracer in both mice and rats.

## Conclusion

In order to establish transgenic tau rodent models as a translational platform for PET imaging research in neurodegenerative disorders, we have developed a range of novel compounds based on the PBB3 structure. One of these compounds, LM229, is shown to have high binding affinity to recombinant tau fibrils. Further evaluation of LM229 showed it to bind to pathological tau inclusions in transgenic human P301S tau mouse brains, with selectivity for tau inclusions in PSP and AD, dementias with different fibrillar tau isoform compositions. In addition, LM229 and PBB3 PET compounds were both shown to bind to tau pathology in brain tissue from two transgenic tau rat models with either 3R and 4R tau inclusions with preferential binding toward the 3R tau inclusions. To our knowledge, this is the first time any tau PET imaging compounds have been shown to bind to tau inclusions in a transgenic rat model. Finally, we have established that LM229 can be used for *in vivo* translational PET research, by developing a method for radiolabeling with the carbon-11 radioisotope and confirmation of BBB entry in mice.

## Methods

### General Methods and Materials

All reagents and solvents were used as supplied or purified using standard procedures as necessary. Analytical separation was conducted on an Ultimate 3000 HPLC system (Thermo Scientific). [^11^C]CO_2_ was produced *via* the ^14^N(p,α) nuclear reaction using GE PETtrace cyclotron applying 16.5 MeV protons onto 0.5% oxygen in nitrogen gas. [^11^C]Methyl iodide was synthesized on a GE Medical Systems MeI MicroLab. A F-4500 FL spectrophotometer (Chemical Engineering department, University of Cambridge) was used to do fluorescence scan of all of the compounds mixed with tau fibrils. Excitation wavelength was 365 nm, and emission was scanned from 400 to 600 nm. With parameters, setting excitation slit was 5.0 nm, emission slit was 20.0 nm, PMT voltage was 950 V, and response was 0.5 s.

All mouse studies were carried out in the Laboratory of Molecular Imaging or the Clifford Allbutt Building, University of Cambridge, and the MRC Laboratory of Molecular Biology. P301S tau transgenic mice^18^ and WT mice were on a pure C57Bl/6 Jax background. The SHR24, SHR72, and SHR wild-type rat brain slices were developed and obtained from AXON Neuroscience.^28^ PSP brain tissue was obtained from the Cambridge Brain Bank, with the PSP cases being part of the cohort created by Prof. James Rowe. AD brain tissue was supplied by the London Neurodegenerative Diseases Brain Bank and Brains for Dementia Research.

#### Preparation of tau fibrils

Monomeric tau (tau-441 human) lyophilized powder was obtained from Eurogentec. Tau monomers (100 μM) were prepared in 20 mM BES buffer (pH 7.4 with 25 mM NaCl and 2 mM dithiothreitol and incubated at 56 °C for 10 min. After being cooled to room temperature in a water bath, heparin (25 μM) and a protease inhibitor mix were added and incubated at 37 °C for 10 days.

#### *In Vitro* and *Ex Vivo* Fluorescence Microscopy

Mice were perfused with PBS followed by 4% PFA. Following 24 h in 4% PFA, the brains were transferred to 20% sucrose prior to being embedded in OCT for cryo-sectioning. Sections (14 μm) were incubated with 50 μM LM229 or PBB3 for 1 h, washed in PBS + 0.1% Triton X-100, and cover-slipped with Vectashield mounting medium. Sections were imaged using a Leica DM6000 microscope. Mouse monoclonal AT8 (MN1020) or AT100 (MN1060) were from ThermoFisher and used at 1:1000. Anti-mouse secondary antibody was conjugated to AlexaFluor647.

### *Ex Vivo* Mice Studies

Mice were injected intravenously (i.v.) with 50 μM LM229 or PBB3 (DMSO) diluted in 50:50 ethanol/PBS. Animals were then perfused (4% PFA) 1 h post-i.v.

### Rat Brain

Fresh frozen rat brain coronal sections (10 μm) were stored on slides at −80 °C until use. Sections were fixed in ice cold acetone for 10 min and air-dried and then rehydrated twice for 5 min with buffer (10 mM PBS with 0.1% Triton X-100) before application of 5% bovine serum albumin for 1 h and two more 5 min buffer washes. Primary monoclonal antibody AT8 (Thermo MN1020, 1:1000) was added overnight at 4 °C, followed by 1 h incubation with a mixture of secondary AlexaFluor555 goat anti-mouse antibody (1:1000) and 50 μM PBB3 or LM229 in 50:50 ethanol/buffer. Following two 5 min washes in buffer, the slides were air-dried and cover-slipped using Fluorsave before analysis. The green autofluorescence of PBB3 and LM229 was visualized using a 488 nm filter and fluorescence of AT8 using a 568 nm filter on a Leica DM6000 microscope.

### DRG Neurons

Neurons were prepared from phenotypic end stage 5–7 month old P301S tau mice and cultured as described previously.^24^ For live staining, neurons cultured for 2 days were washed in PBS and incubated with various concentrations of LM229 for 20 min at room temperature. After three washes in PBS, neurons were imaged on a Leica DMI 4000B microscope using a DFC3000 G camera and application suite 4.0.0.11706. The dead cell stain Propidium iodide (1 μg/mL) was added to ensure that only live cells were imaged. Phase contrast images were taken to show viable neurons. For fixed cell staining, neurons were fixed for 20 min at room temperature in 4% paraformaldehyde, washed in PBS, and permeabilized in PBS containing 0.3% Triton X-100. LM299 was added as described for live cells, and cells were probed with AT100 (1:1000, MN1060) followed by anti-mouse AlexaFluor568 secondary antibody. Cultures were co-stained with the nuclear dye DAPI (blue) to reveal total cells.

### Radiosynthesis of [^11^C]LM229

TBS-protected precursor **E** (1.5 mg) in DMSO (150 μL) was added to a suspension of powdered KOH (15 mg) in DMSO (300 μL), and the suspension mixed *via* vortex for 1 min. [^11^C]Methyl iodide was trapped in the suspension and heated to 125 °C for 5 min. Water (200 μL) was then added and the mixture heated to 60 °C for 2 min. The mixture was diluted with water (0.8 mL), and the radioactive material was loaded into a preparative HPLC system for purification (42% acetonitrile/50 mM ammonium formate, 6 mL/min, Luna 5μ, 10.00 mm internal diameter × 250 mm (Phenomenex)). The fraction corresponding to [^11^C]LM229 was collected in water (100 mL) and reformulated into saline (6 mL) containing ethanol (400 μL) and 25% ascorbic acid (200 μL) using a C18-light sep-pak cartridge to afford [^11^C]LM229 (1.0–1.4 GBq, 10% DCY). Analytical HPLC (Luna 5μ, 4.60 mm internal diameter × 250 mm (Phenomenex), 50% acetonitrile/50 mM ammonium formate, 1 mL/min) confirmed the formulated product was radiochemically pure (>95%). The specific activity of [^11^C]LM229 was >200 GBq/μmol.

### PET Studies with [^11^C]LM229

These studies were regulated under the Animals (Scientific Procedures) Act 1986 Amendment Regulations 2012 following ethical review by the University of Cambridge Animal Welfare and Ethical Review Body (AWERB). The mice were anesthetized with 5% isoflurane, and general anesthesia was maintained using 1.5% isoflurane. The anesthetized mice were placed prone on the bed of a microPET Focus-220 scanner (Concorde Microsystems, Knoxville, TN). Body temperature was maintained at 37 °C using a heating blanket connected to a rectal thermistor probe. Blood oxygen saturation as well as heart rate and breathing rate were monitored and maintained within normal limits using a noninvasive mouseOX pulse-oximeter sensor (Starr Life Science Corp., Oakmont, PA) attached to the thigh. Before the injection of a tracer, a transmission scan was performed with a 68Ge point source for attenuation and scatter correction of 511 keV photons. [^11^C]LM229 was injected *via* the tail vein over 30 s, followed by a 15 s heparin–saline flush. Dynamic data were acquired in list mode for 90 min. Data were subsequently Fourier rebinned in following time frames: 12 × 5 s, 6 × 10 s, 3 × 20 s, 4 × 30 s, 5 × 60 s, 10 × 120 s, 12 × 5 min. Corrections were applied for randoms, dead time, normalization, attenuation, and decay. Fourier rebinning was used to compress the 4D sinograms to 3D before reconstruction with a 2D-filtered back projection with a Hann window cutoff at the Nyquist frequency. The image voxel size was 0.95 × 0.95 × 0.80 mm, with an array size of 128 × 128 × 95.

Three-dimensional volumes of interest (VOIs) for mice brain MRI template available on PMOD software (version 3.8; PMOD technologies, Zurich, Switzerland) were modified to obtain a whole brain VOI. Individual PET images were then co-registered with this MRI template, and the VOI transferred from MRI to PET. Whole-brain time–activity curves were obtained for each of the animals. The results were expressed as % injected dose per gram, assuming a specific gravity of 1 g·mL^–1^ for brain tissue.

## Supporting Information Available

Synthetic and analytical data of compounds, immunohistochemistry, and binding affinity methods (PDF)

## Abbreviations

PET: positron emission tomography
Aβ: β-amyloid
AD: Alzheimer’s disease
PSP: progressive supranuclear palsy
PiB: Pittsburgh Compound B
NFT: neurofibrillary tangle
DTT: dithiothreitol
4R: four repeat tau
3R: three repeat tau
