# Supplementary material for: Synthesis and Assessment of Novel Probes for Imaging Tau Pathology in Transgenic Mouse and Rat Models
Source: ACS Chem Neurosci. 2021 Mar 10;12(11):1885–93. doi: 10.1021/acschemneuro.0c00790 (PMC8176454; doi:10.1021/acschemneuro.0c00790)

Supporting Information

Synthesis and Assessment of Novel Probes for Imaging Tau Pathology in Transgenic Mouse and Rat Models.

Lindsay McMurray[1], Jennifer A. Macdonald[2,] Nisha Kuzhuppilly Ramakrishnan[1], Yanyan Zhao[1], David W. Williamson[1], O. Tietz[1], Xiaoyun Zhou[1], Steven Kealey[1], Steven G. Fagan[3], Tomáš Smolek[4], Veronika Cubinkova[4], Norbert Žilka[4], Maria Grazia Spillantini[3], Aviva M Tolkovsky[3] , Michel Goedert[2], Franklin I. Aigbirhio[1,3*]

[1]Molecular Imaging Chemistry Laboratory, Wolfson Brain Imaging Centre, University of Cambridge, Cambridge, UK.

[2]MRC Laboratory of Molecular Biology, Cambridge, UK.

[3]Department of Clinical Neurosciences, University of Cambridge, Cambridge, UK

[4]Axon Neuroscience R&D Services SE, Bratislava, Slovak Republic

General Procedures.

Data collection: Proton nuclear magnetic resonance (^{1}H NMR) spectra were recorded at ambient temperature, unless otherwise stated, on a Bruker AM 300 (300 MHz) or an Advance 500 (500 MHz) spectrometer. Chemical shifts are reported relative to residual protons in $CDCl_3$ (δ_H 7.26 ppm) or DMSO-d_6 (δ_H 2.50 ppm) and coupling constants (*J*) are given in Hz. Data are reported as follows: chemical shift (number of protons, multiplicity, coupling constants). Chemical shift was measured in ppm and quoted to the nearest 0.01 ppm. Coupling constants were quoted to the nearest 0.1 Hz and multiplicity reported according to the following convention: s = singlet, d = doublet, t = triplet, q = quartet, qn = quintet, m = multiplet, br = broad. Carbon nuclear magnetic resonance (^{13}C NMR) spectra were recorded at ambient temperature with broadband proton decoupling on a Bruker AM 300 (75 MHz) or an Advance 500 (125 MHz) spectrometer. Chemical shift was measured in ppm and quoted to the nearest 0.1 ppm relative to an internal reference: $CDCl_3$ (δ_C 77.2 ppm) or DMSO-d_6 (δ_H 40.0 ppm). High resolution mass spectra were made on a Micromass Q-TOF spectrometer using EI (electron impact) or ES (electrospray ionization) techniques by the EPSRC mass spectrometry service (Swansea).

Chromatography: Analytical thin layer chromatography was performed using pre-coated Alugram aluminium backed silica gel plates (Silicagel 60). Visualization was by ultraviolet fluorescence (λ = 254 nm). Flash column chromatography was carried out on Material Harvest Silica gel 60 (230-400 mesh) under positive pressure.

Solvents and reagents: All reagents and solvents were used as supplied or purified using standard procedures as necessary. Aqueous solutions are saturated unless otherwise stated. Where indicated reactions were carried out under an atmosphere of nitrogen.

General Procedures

General Procedure A: To a solution of the appropriate phosphonate (1.2 equiv.) in tetrahydrofuran at -78 ˚C was added lithium bis(trimethylsilyl)amide (1M solution in tetrahydrofuran, 1.4 equiv.). The solution was stirred at room temperature for 1 h after which the solution was cooled to -78 ˚C and a solution of the appropriate

aldehyde (1.0 equiv.) in tetrahydrofuran was added. The solution was warmed to room temperature and stirred for 16 h. Acetic acid was then added until the solution reached pH 7 followed by addition of water. The aqueous layer extracted with ethyl acetate and the combined organic layers were washed with saturated aqueous sodium bicarbonate, dried (Na_2SO_4) and concentrated *in vacuo*. The crude product was purified by flash column chromatography to afford the desired compound.

General Procedure B for TBS deprotection: To a solution of the appropriate precursor (1.0 equiv.) in tetrahydrofuran was added pyridine and HF-pyridine and the solution stirred at room temperature for 1 h before being quenched by addition of saturated aqueous sodium bicarbonate solution. The aqueous layer was extracted with ethyl acetate and the combined organics were washed with water, dried (Na_2SO_4) and concentrated *in vacuo*. The crude product was purified by recrystallization from water-ethanol to afford the desired compound.

Experimental data

(*E*)-methyl 3-(6-((tert-butoxycarbonyl)amino)pyridin-3-yl)acrylate

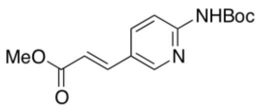

To a solution of methyl (2*E*)-3-(6-aminopyridin-3-yl)prop-2-enoate (1.10 g, 6.18 mmol) in dioxane (30 mL) was added triethylamine (912 µL, 6.59 mmol), 4-dimethylaminopyridine (253 mg, 2.07 mmol) and di-*tert*-butyl dicarbonate (1.56 g, 6.80 mmol) and the reaction stirred at room temperature for 16 h, after which the reaction mixture was concentrated *in vacuo*. The crude product was purified by recrystallization from ethyl acetate to afford the title compound (1.20 g, 4.00 mmol, 65%)

^{1}H NMR (300 MHz, $CDCl_3$) δ_H 8.39 (1H, d, *J* = 2.5 Hz), 8.28 (1H, br s), 8.04 (1H, d, *J* = 8.7 Hz), 7.87 (1H, dd, *J* = 8.7, 2.5 Hz), 7.62 (1H, d, *J* = 15.9 Hz), 6.40 (1H, d, *J* = 15.9 Hz), 3.81 (3H, s), 1.55 (9H, s); **^{13}C NMR** (75 MHz, $CDCl_3$) δ_C 167.1, 153.2, 152.0, 148.0, 140.7, 136.6, 125.1, 117.6, 112.3, 81.7, 51.8, 28.2; **m/z** HRMS (ESI) found [M+H]+, 301.1160, $C_{14}H_{18}N_2O_4Na$ requires 301.1159

(*E*)-*tert*-butyl (5-(3-oxoprop-1-en-1-yl)pyridin-2-yl)carbamate

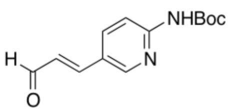

Step 1: To a solution of (*E*)-methyl 3-(6-((tert-butoxycarbonyl)amino)pyridin-3-yl)acrylate (1.40 g, 5.03 mmol) in dichloromethane (60 mL) at -78 ˚C was added diisobutylaluminium hydride (1 M solution in tetrahydrofuran, 10.1 mL) and the reaction mixture stirred at -78 ˚C for 30 min followed by room temperature for 1.5 h. The reaction mixture was cooled to -78 ˚C and further diisobutylaluminium hydride (1 M solution in tetrahydrofuran, 5.00 mL) was added. The reaction mixture was then warmed to room temperature and stirred for a further 2 h. The solution was cooled to -78 ˚C and methanol (1.0 mL) was added. The solution was warmed to room temperature and aqueous saturated Rochelle salts solution (100 mL) was added and the resulting solution stirred at room temperature for 1 h after which the aqueous layer was extracted with dichloromethane. The combined organic layers were dried ($MgSO_4$) and concentrated *in vacuo*.

Step 2: The crude alcohol from step 1 was dissolved in tetrahydrofuran (40 mL) and activated manganese dioxide (4.42 g, 50.8 mmol) was added. The reaction mixture was stirred at room temperature for 16 h after which the mixture was filtered. The filtrate was concentrated *in vacuo*. The crude product was purified by flash column chromatography (40-50% ethyl acetate/petroleum ether) to afford the title compound (1.06 g, 4.27 mmol, 85%)

^{1}H NMR (300 MHz, $CDCl_3$) δ_H 9.70 (1H, d, J = 7.6 Hz), 8.44 (1H, d, J = 2.0 Hz), 8.18 (1H, br s), 8.09 (1H, d, J = 8.9 Hz), 7.90 (1H, dd, J = 8.9, 2.0 Hz), 7.41 (1H, d, J = 16.0 Hz), 6.69 (1H, dd, J = 16.0, 7.6 Hz), 1.56 (9H, s); **^{13}C NMR** (75 MHz, $CDCl_3$) δ_C 193.1, 154.0, 151.9, 148.7, 148.4, 136.9, 128.1, 124.8, 112.4, 81.9, 28.2; **m/z** HRMS (ESI) found [M+H]+, 249.1236, $C_{13}H_{16}N_2O_3$ requires 249.1234

Diethyl ((6-((*tert*-butyldimethylsilyl)oxy)benzo[d]thiazol-2-yl)methyl)phosphonate

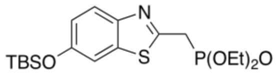

To a solution of lithium diisopropylamide (2 M solution in THF/heptane/ethylbenzene, 1.66 mL) in tetrahydrofuran (2.5 mL) at -78 ˚C was added a solution of 6-((*tert*-butyldimethylsilyl)oxy)-2-methylbenzo[d]thiazole (440 mg, 1.58 mmol) in tetrahydrofuran (4 mL). The solution was stirred at -78 ˚C for 30 min followed by room temperature for 15 min. The solution was then cooled to -78 ˚C and diethyl chlorophosphonate (252 µL, 1.74 mmol) was added. The solution was stirred at -78 ˚C for 10 min followed by room temperature for 16 h. Saturated aqueous ammonium chloride (10 mL) was added and the aqueous layer extracted with ethyl acetate. The combined organic layers were washed with saturated aqueous sodium bicarbonate (30 mL) and water (30 mL), dried (Na_2SO_4) and concentrated *in vacuo*. The crude product was purified by flash column chromatography (2–5% methanol/dichloromethane) to afford the title compound (400 mg, 0.964 mmol, 61%).

^{1}H NMR (300 MHz, $CDCl_3$) δ_H 7.80 (1H, d, J = 9.0 Hz), 7.25 (1H, d, J = 2.5 Hz), 6.95 (1H, dd, J = 9.0, 2.5 Hz), 4.14 (2H, q, J = 7.0 Hz), 4.12 (2H, q, J = 7.0 Hz), 3.70 (1H, s), 3.63 (1H, s), 1.30 (3H, t, J = 7.0 Hz), 1.29 (3H, t, J = 7.0 Hz), 0.98 (9H, s), 0.20 (6H, s); **^{13}C NMR** (75 MHz, $CDCl_3$) δ_C 158.6, 158.4, 153.5, 147.8, 136.9, 123. 1, 119.8, 111.4, 62.8, 62.7, 33.8, 31.9, 25.5, 18.1, 16.3, 16.2, -4.5; **m/z** HRMS (ESI) found [M+H]+, 416.1474, $C_{18}H_{30}NO_4PSSi$ requires 416.1475

***Tert*-butyl (5-((1*E*, 3*E*)-4-(6-((*tert*-butyldimethylsilyl)oxy)benzo[d]thiazol-2-yl)buta-1,3-dien-1-yl)pyridin-2-yl)carbamate**

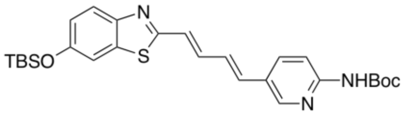

To a solution of diethyl ((6-((*tert*-butyldimethylsilyl)oxy)benzo[d]thiazol-2-yl)methyl)-phosphonate (252 mg, 0.606 mmol) in tetrahydrofuran (1.0 mL) at -78 ˚C was added lithium bis(trimethylsilyl)amide (1M solution in tetrahydrofuran, 582 µL). The solution was stirred at room temperature for 1 h after which the solution was cooled to -78 ˚C and a solution of (*E*)-*tert*-butyl (5-(3-oxoprop-1-en-1-yl)pyridin-2-yl)carbamate (100 mg, 0.404 mmol) in tetrahydrofuran (2 mL) was added. The solution was warmed to room temperature and stirred for 4 h. Acetic acid was then added until the solution reached pH 7 followed by addition of water. The aqueous layer was extracted with ethyl acetate and the combined organic layers were washed with saturated aqueous sodium

bicarbonate, dried (Na_2SO_4) and concentrated *in vacuo*. The crude product was purified by recrystallization from ethyl acetate to afford the title compound (155 mg, 0.305 mmol, 75%)

^{1}H NMR (300 MHz, $CDCl_3$) δ_H 8.31 (1H, d, *J* = 2.1 Hz), 8.16 (1H, br s), 7.9 (1H, d, *J* = 8.8 Hz), 7.83 (1H, dd, *J* = 8.6, 2.4 Hz), 7.81 (1H, d, *J* = 8.8), 7.17 – 7.26 (2H, m), 6.92 – 6.98 (2H, m), 6.91 (1H, d, *J* = 15.2 Hz), 6.75 (1H, d, *J* = 15.7 Hz), 1.54 (12H, s), 1.00 (9H, s), 0.23 (6H, s); **^{13}C NMR** (75 MHz, $CDCl_3$) δ_C 164.6, 153.9, 152.2, 151.7, 148.9, 146.8, 136.6, 135.6, 135.4, 132.7, 127.5, 127.4, 125.8, 123.3, 120.2, 112.3, 111.6, 81.3, 28.3, 25.6, 18.2, -4.4; **m/z** HRMS (ESI) found [M+H]+, 510.2230 $C_{27}H_{36}N_3O_3SSi$ requires 510.2241

5-((1*E*,3*E*)-4-(6-((*tert*-butyldimethylsilyl)oxy)benzo[d]thiazol-2-yl)buta-1,3-dien-1-yl)pyridin-2-amine

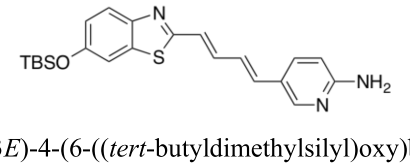

To a solution of *tert*-butyl (5-((1*E*, 3*E*)-4-(6-((*tert*-butyldimethylsilyl)oxy)benzo[d]thiazol-2-yl)buta-1,3-dien-1-yl)pyridin-2-yl)carbamate (100 mg, 0.982 mmol) in dichloromethane (2.0 mL) was added anisole (272 µL, 2.51 mmol) followed by trifluoroacetic acid (936 µL, 12.2 mmol) and the solution was stirred at room temperature for 16 h. Saturated aqueous sodium bicarbonate was added and the aqueous layer extracted with ethyl acetate. The combined organic layers were dried (Na_2SO_4) and concentrated *in vacuo*. The crude product was purified by recrystallization from water-ethanol (5:1) to afford the title compound (340 mg, 0.831 mmol, 85%)

^{1}H NMR (300 MHz, $CDCl_3$) δ_H 8.04 (1H, d, *J* = 2.3 Hz), 7.77 (1H, dd, *J* = 8.7 Hz), 7.68 (1H, dd, *J* = 8.7, 2.3 Hz), 7.52 (1H, d, *J* = 2.3 Hz), 7.27 (1H, dd, *J* = 15.1, 8.7 Hz), 6.91 – 7.00 (2H, m), 6.86 (2H, d, *J* = 15.9 Hz), 6.47 (1H, d, *J* = 8.7 Hz), 6.36 (2H, s); **^{13}C NMR** (75 MHz, $CDCl_3$) δ_C 165.3, 160.3, 153.4, 149.2, 149.1, 138.8, 135.8, 135.6, 134.6, 123.7, 123.3, 121.3, 120.3, 112.5, 108.8, 26.0, 18.4, -4.1; **m/z** HRMS (ESI) found [M+H]+, 410.1715, $C_{22}H_{27}N_3OSSi$ requires 410.1715

(*E*)-methyl 3-(6-(methylamino)pyridin-3-yl)acrylate

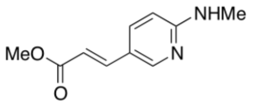

Step 1: To a solution of (*E*)-methyl 3-(6-((tert-butoxycarbonyl)amino)pyridin-3-yl)acrylate (280 mg, 1.01 mmol) in dimethylformamide (5.0 mL) at 0 ˚C was added sodium hydride (60% suspension in mineral oil, 52 mg, 1.30 mmol). The suspension was stirred for 20 min at 0 ˚C followed by dropwise addition of methyl iodide (72 µL, 1.16 mmol). The reaction mixture was stirred at 0 ˚C for 20 min then warmed to room temperature over 1 h. Water was added and the aqueous layer extracted with ethyl acetate. The combined organic layers were washed with water, 0.1 M hydrochloric acid, saturated aqueous sodium bicarbonate, and brine, dried (Na_2SO_4) and concentrated *in vacuo*.

Step 2: The crude product from Step 1 was dissolved in dichloromethane (25 mL) and cooled to 0˚C followed by addition of trifluoroacetic acid (10 mL). The solution was warmed to room temperature and stirred for 16 h before being cooled to 0 ˚C and quenched by addition of saturated aqueous sodium bicarbonate. The aqueous layer extracted with ethyl acetate and the combined organic layers dried ($MgSO_4$) and concentrated *in vacuo*. The crude product was purified by flash column chromatography (50% ethyl acetate/petroleum ether) to afford the title compound (140 mg, 0.729 mmol, 72%)

^{1}H NMR (300 MHz, $CDCl_3$) δ_H 8.22 (1H, d, *J* = 2.2 Hz), 7.66 (1H, dd, *J* = 8.7, 2.2 Hz), 7.6 (1H, d, *J* = 16.2 Hz), 6.41 (1H, d, *J* = 8.7 Hz), 6.23 (1H, d, *J* = 16.2 Hz), 4.87 (1H, br s), 3.79 (3H, s), 2.98 (3H, d, *J* = 5.2 Hz); **^{13}C NMR** (75 MHz, $CDCl_3$) δ_C 167.7, 159.8, 149.3, 141.9, 135.8, 119.6, 113.9, 106.8, 51.6, 29.0; **m/z** HRMS (ESI) found [M+H]+, 193.0970, $C_{10}H_{13}N_2O_2$ requires 193.0972

(*E*)-3-(6-(methylamino)pyridin-3-yl)acrylaldehyde

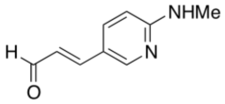

Step 1: To a solution of (E)-methyl 3-(6-(methylamino)pyridin-3-yl)acrylate (130 mg, 0.677 mmol) in dichloromethane (10 mL) at -78 ˚C was added diisobutylaluminium hydride (1 M solution in tetrahydrofuran, 2.04 mL) and the reaction mixture stirred at -78 ˚C for 30 min followed by room temperature for 1.5 h. The reaction mixture was cooled to -78 ˚C and further diisobutylaluminium hydride (1 M solution in tetrahydrofuran, 1.00 mL) was added. The reaction mixture was then warmed to room temperature and stirred for a further 2 h. The solution was cooled to -78 ˚C and methanol (1.00 mL) was added. The solution was warmed to room temperature and aqueous saturated Rochelle salts solution (100 mL) was added and the resulting solution stirred at room temperature for 1 h after which the aqueous layer was extracted with dichloromethane. The combined organic layers were dried ($MgSO_4$) and concentrated *in vacuo*.

Step 2: The crude alcohol from step 1 was dissolved in tetrahydrofuran (5 mL) and activated manganese dioxide (590 mg, 6.78 mmol) was added. The reaction mixture was stirred at room temperature for 16 h after which the mixture was filtered. The filtrate was concentrated *in vacuo*. The crude product was purified by flash column chromatography (70% ethyl acetate/petroleum ether) to afford the title compound (95 mg, 0.586 mmol, 87%).

^{1}H NMR (300 MHz, $CDCl_3$) δ_H 8.23 (1H, d, *J* = 2.3 Hz), 7.66 (1H, dd, *J* = 8.9, 2.3 Hz), 7.35 (1H, d, *J* = 15.8 Hz), 6.51 (1H, dd, *J* = 15.8, 7.7 Hz), 6.43 (1H, d, *J* = 8.9 Hz), 5.26 (1H, br s), 2.98 (3H, d, *J* = 5.1 Hz); **^{13}C NMR** (75 MHz, $CDCl_3$) δ_C 193.4, 160.7, 151.2, 150.5, 135.5, 124.6, 119.3, 106.9, 28.8; **m/z** HRMS (ESI) found [M+H]+, 163.0863 $C_9H_{11}N_2O_2$ requires 163.0863

5-((1*E*,3*E*)-4-(6-((*tert*-butyldimethylsilyl)oxy)benzo[d]thiazol-2-yl)buta-1,3-dien-1-yl)-N-methylpyridin-2-amine

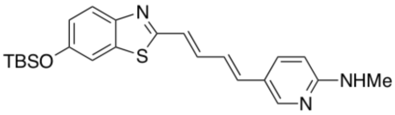

To a solution of diethyl ((6-((*tert*-butyldimethylsilyl)oxy)benzothiazol-2-yl)methyl)-phosphonate (500 mg, 1.20 mmol) in tetrahydrofuran (1.5 mL) at -78 ˚C was added lithium bis(trimethylsilyl)amide (1M solution in tetrahydrofuran, 1.16 mL). The solution was stirred at room temperature for 1 h after which the solution was cooled to -78 ˚C and a solution of (*E*)-3-(6-(methylamino)pyridin-3-yl)acrylaldehyde (130 mg, 0.802 mmol) in tetrahydrofuran (4 mL) was added. The solution was warmed to room temperature and stirred for 16 h. Acetic acid was then added until the solution reached pH 7 followed by addition of water. The aqueous layer extracted with ethyl acetate and the combined organic layers were washed with saturated aqueous sodium bicarbonate, dried (Na_2SO_4) and concentrated *in vacuo*. The crude product was purified by flash column chromatography (50-80% ethyl acetate/petroleum ether to afford the title compound (210 mg, 0.496 mmol, 62%).

^{1}H NMR (300 MHz, DMSO-d$_6$) δ_H 8.14 (1H, d, *J* = 2.2 Hz), 7.79 (1H, d, *J* = 8.8 Hz), 7.67 (1H, dd, *J* = 9.2, 2.2 Hz), 7.16 – 7.26 (2H, m), 6.96 (1H, dd, *J* = 8.8, 2.6 Hz), 6.70 – 6.97 (3H, m), 6.42 (1H, d, *J* = 8.8 Hz), 4.84 – 4.89 (1H, m), 2.97 (3H, d, *J* = 5.4 Hz), 1.01 (9H, s), 0.24 (6H, s; **^{13}C NMR** (75 MHz, DMSO-d$_6$) δ_C 165.1, 159.1, 153.7, 149.0, 148.2, 137.6, 135.4, 134.5, 134.2, 124.3, 124.0, 123.0, 122.0, 120.0, 111.6, 106.6, 29.0, 25.6, 18.2, -4.4; **m/z** HRMS (ESI) found [M+H]+, 424.1870, $C_{23}H_{30}N_3OSSi$ requires 424.1873

PBB3

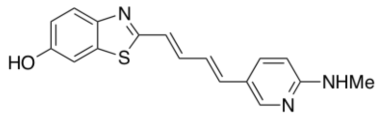

To a solution of ((6-((*tert*-butyldimethylsilyl)oxy)benzothiazol-2-yl)methyl)-phosphonate (110 mg, 0.260 mmol) in tetrahydrofuran (500 µL) was added pyridine (250 µL) followed by HF-pyridine (250 µL) and the solution was stirred at room temperature for 2 h. Saturated aqueous sodium bicarbonate was added and the aqueous layer extracted with ethyl acetate. The combined organic layers were dried (Na_2SO_4) and concentrated *in vacuo*. The crude product was purified by recrystallization from water-ethanol (5:1) to afford the title compound (40 mg, 0.129 mmol, 50%).

^{1}H NMR (300 MHz, DMSO-d$_6$) δ_H 9.84 (1H, s), 8.09 (1H, d, *J* = 2.3 Hz), 7.70 (1H, d, *J* = 9.2 Hz), 7.69 (1H, dd, *J* = 8.8, 2.3 Hz), 7.32 (1H, d, *J* = 2.3 Hz), 7.22 (1H, dd, *J* = 15.2, 9.7 Hz), 6.81 – 6.98 (5H, m), 6.48 (1H, d,d *J* = 8.8 Hz), 2.80 (3H, d, *J* = 4.6 Hz); **^{13}C NMR** (75 MHz, DMSO-d$_6$) δ_C 163.5, 159.7, 149.0, 147.6, 138.2, 135.8, 135.4, 133.9, 123.8, 123.4, 123.4, 121.0, 116.3, 108.7, 107.1, 28.4; **m/z** HRMS (ESI) found [M+H]+, 310.1011, $C_{17}H_{16}N_3OS$ requires 310.1009

N-(3-fluoro-4-methoxyphenyl)acetamide

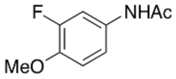

To a solution of acetyl chloride (2.56 mL, 36.1 mmol) in CH_2Cl_2 (15 mL) at 0 ˚C was added 3-fluoro-*p*-anisidine (5.00 g, 35.0 mmol) and triethylamine (4.97 mL, 35.7 mmol) in CH_2Cl_2 (15 mL). The solution was stirred at 0 ˚C for 1 h followed by room temperature for 18 h after which the solution was basified with 20% aqueous K_2CO_3 solution. The aqueous layer extracted with CH_2Cl_2 and dried ($MgSO_4$) and concentrated *in vacuo*. The crude product was purified by flash column chromatography (50-60% ethyl acetate/petroleum ether) to afford the title compound (4.24 g, 35.00 mmol, quant.).

^{1}H NMR (300 MHz, $CDCl_3$) δ_H 7.53 (1H, br s), 7.40 (1H, dd, *J* = 12.8, 2.6 Hz), 7.10 – 7.14 (1H, m), 6.88 (1H, t, *J* = 8.9 Hz), 3.86 (3H, s), 2.15 (3H, s); **^{13}C NMR** (75 MHz, $CDCl_3$) δ_C 168.4, 152.1 (d, *J* = 245 Hz), 144.4 (d, *J* = 9.8 Hz), 131.3 (d, *J* = 9.8 Hz), 115.8 (d, *J* = 3.5 Hz), 113.6 (d, *J* = 2.8 Hz), 109.3 (d, *J* = 22.4 Hz), 56.5, 24.3; **^{19}F NMR** (282 MHz, $CDCl_3$) δ_F -133.2 (dd, *J* = 12.8, 8.9 Hz); **m/z** HRMS (ESI) found [M+H]+, 184.0765, $C_9H_{11}NO_2F$ requires 184.0757

N-(5-fluoro-2-iodo-4-methoxyphenyl)acetamide

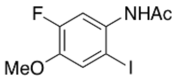

To a solution of *N*-(3-fluoro-4-methoxyphenyl)acetamide (2.60 g, 21.5 mmol) in acetonitrile (25 mL) was added iodine (2.73 g, 21.5 mmol) and sodium *meta* periodate (1.37 g, 6.40 mmol) and the solution was heated to 70 ˚C. Once 70 ˚C was reached concentrated sulfuric acid (632 µL) in acetonitrile (1.3 mL) was added dropwise over 15 min. The solution was then allowed to stir at 70 ˚C for 4 h before being cooled to room temperature and quenched with 25% aqueous Na_2SO_3 solution. The aqueous layer extracted with ethyl acetate and the combined organics were dried (Na_2SO_4) and concentrated *in vacuo*. The crude product was purified by flash column chromatography (30-50% ethyl acetate/petroleum ether) to afford the title compound (2.65 g, 8.95 mmol, 42%)
^{1}H NMR (300 MHz, $CDCl_3$) δ_H 8.02 (1H, d, J = 13.3 Hz), 7.31 (1H, d, J = 8.5 Hz), 7.25 (1H, br s), 3.86 (3H, s), 2.23 (3H, s); **^{13}C NMR** (75 MHz, $CDCl_3$) δ_C 168.1, 152.24 (d, J = 247.7 Hz), 145.0 (d, J = 11.5 Hz), 132.0 (d, J = 9.6 Hz), 122.7 (d, J = 2.3 Hz), 110.8 (d, J = 23.4 Hz), 82.1, 56.7, 24.5; **^{19}F NMR** (282 MHz, $CDCl_3$) δ_F -131.1 (dd, J = 13.3, 8.5 Hz); **m/z** HRMS (ESI) found [M+H]+, 309.9735, $C_9H_{10}NO_2FI$ requires 309.9735

5-fluoro-6-methoxy-2-methylbenzo[d]thiazole

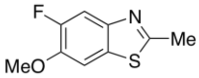

To a mixture of *N*-(5-fluoro-2-iodo-4-methoxyphenyl)acetamide (2.54 g, 8.22 mmol), copper(I) iodide (163 mg, 0.858 mmol), and sodium sulfide nonahydrate (6.19 g, 25.8 mmol) was added dimethylformamide (20 mL) and the resulting reaction mixture was heated to 80 ˚C for 18 h. The solution was then cooled to room temperature and concentrated hydrochloric acid (6.70 mL) was added. After stirring at room temperature for 7 h, saturated aqueous sodium bicarbonate solution was added and the aqueous layer was extracted with ethyl acetate. The combined organics were washed with water and brine, dried (Na_2SO_4) and concentrated *in vacuo*. The crude product was purified by flash column chromatography (10% ethyl acetate/petroleum ether) to afford the title compound (1.20 g, 6.08 mmol, 74%).
^{1}H NMR (300 MHz, $CDCl_3$) δ_H 7.65 (1H, d, J = 11.6 Hz), 7.34 (1H, d, J = 7.9 Hz), 3.96 (3H, s), 2.81 (3H, s); **^{13}C NMR** (75 MHz, $CDCl_3$) δ_C 166.3, 152.2 (d, J = 245.0 Hz), 146.8, 146.6 (d, J = 13.6 Hz), 130.9 (d, J = 2.3 Hz), 109.0 (d, J = 21.2 Hz), 104.3 (d, J = 2.6 Hz), 56.6, 19.9; **^{19}F NMR** (282 MHz, $CDCl_3$) δ_F -135.77 (dd, J = 11.6, 7.9 Hz); **m/z** HRMS (ESI) found [M+H]+, 198.0381, C_9H_8FNOS requires 198.0383

5-fluoro-2-methylbenzo[d]thiazol-6-ol

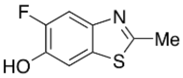

To a solution of 5-fluoro-6-methoxy-2-methylbenzo[d]thiazole (500 mg, 2.54 mmol) in dichloromethane (60 mL) was added boron tribromide (5.08 mL, 1 M solution in dichloromethane) and the solution was stirred at 50 ˚C for 18 h. The cooled reaction mixture was quenched with saturated aqueous sodium bicarbonate solution and the aqueous layer extracted with dichloromethane. The combined organics were dried ($MgSO_4$) and concentrated *in vacuo*. The crude product was purified by flash column chromatography (10-20 % ethyl acetate/petroleum ether) to afford the title compound (400 mg, 2.19 mmol, 86%).

^{1}H NMR (300 MHz, $CDCl_3$) δ_H 7.66 (1H, d, *J* = 11.3 Hz), 7.41 (1H, d, *J* = 8.4 Hz), 2.80 (3H, s); **^{13}C NMR** (75 MHz, $CDCl_3$) δ_C 166.8, 146.5 (d, *J* = 9.9 Hz), 151.0 (d, *J* = 237.0 Hz), 142.5 (d, *J* = 17.1 Hz), 131.5 (d, *J* = 1.5 Hz), 108.4 (d, *J* = 21.0 Hz), 108.1 (d, *J* = 2.4 Hz), 19.9; **^{19}F NMR** (282 MHz, $CDCl_3$) δ_F -139.9 (t, *J* = 11.3 Hz); **m/z** HRMS (ESI) found [M+H]+, 184.0227 $C_8H_7FNO_S$ requires 184.0224

6-((*tert*-butyldimethylsilyl)oxy)-5-fluoro-2-methylbenzo[d]thiazole

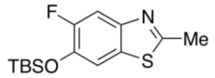

To a solution of 5-fluoro-2-methylbenzo[d]thiazol-6-ol (196 mg, 1.07 mmol) in tetrahydrofuran (5 mL) was added imidazole (145 mg, 2.13 mmol) and *tert*-butyldimethylsilyl chloride (241 mg, 1.60 mmol) and the solution was stirred at room temperature for 16 h. The reaction mixture was quenched with saturated aqueous ammonium chloride solution and the aqueous layer extracted with ethyl acetate. The combined organics were dried (Na_2SO_4) and concentrated *in vacuo*. The crude product was purified by flash column chromatography (10% ethyl acetate/petroleum ether) to afford the title compound (297 mg, 1.00 mmol, 93%).

^{1}H NMR (300 MHz, $CDCl_3$) δ_H 7.62 (1H, d, *J* = 10.8 Hz), 7.30 (1H, d, *J* = 7.9 Hz), 2.78 (3H, s), 1.03 (9H, s), 0.21 (6H, s); **^{13}C NMR** (75 MHz, $CDCl_3$) δ_C 166.7, 153.9 (d, *J* = 243.6 Hz), 147.7 (d, *J* = 11.1 Hz), 142.0 (d, *J* = 15.6 Hz), 130.9 (d, *J* = 2.2 Hz), 112.9 (d, *J* = 2.5 Hz), 109.1 (d, *J* = 22.2 Hz), 25.5, 19.9, 18.3, -4.8; **^{19}F NMR** (282 MHz, $CDCl_3$) δ_F -131.9 (dd, *J* = 10.8, 7.9 Hz); **m/z** HRMS (ESI) found [M+H]+, 298.1088, $C_{14}H_{20}FNOSSi$ requires 298.1092

Diethyl ((6-((tert-butyldimethylsilyl)oxy)-5-fluorobenzo[d]thiazol-2-yl)methyl)phosphonate

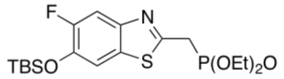

To a solution of lithium diisopropylamide (2 M solution in THF/heptane/ethylbenzene, 1.64 mL) in tetrahydrofuran (2 mL) at -78 ˚C was added a solution of 6-((*tert*-butyldimethylsilyl)oxy)-5-fluoro-2-methylbenzothiazole (320 mg, 1.08 mmol) in tetrahydrofuran (3 mL). The solution was stirred at -78 ˚C for 30 min followed by room temperature for 15 min. The solution was then cooled to -78 ˚C and diethyl chlorophosphonate (234 µL, 1.62 mmol) was added. The solution was stirred at -78 ˚C for 10 min followed by room temperature for 16 h. Saturated ammonium chloride (10 mL) was added and the aqueous layer extracted with ethyl acetate. The combined organic layers were washed with saturated aqueous sodium bicarbonate (30 mL) and water (30 mL), dried (Na_2SO_4) and concentrated *in vacuo*. The crude product was purified by flash column chromatography (70-90% ethyl acetate/petroleum ether) to afford the title compound (236 mg, 0.710 mmol, 66%).

^{1}H NMR (300 MHz, $CDCl_3$) δ_H 7.67 (1H, d, *J* = 10.7 Hz), 7.33 (1H, d, *J* = 8.2 Hz), 4.18 (2H, q, *J* = 7.2 Hz), 4.15 (2H, q, *J* = 7.2 Hz), 3.72 (1H, s), 3.65 (1H, s), 1.33 (6H, t, *J* = 7.2 Hz), 1.03 (9H, s), 0.22 (6H, s); **^{13}C NMR** (75 MHz, $CDCl_3$) δ_C 160.4 (d, *J* = 8.8 Hz), 153.8 (d, *J* = 242.9 Hz), 147.1 (d, *J* = 10.7 Hz), 142.1 (d, *J* = 15.7 Hz), 131.1, 112.6 (d, *J* = 1.8 Hz), 109.2 (d, *J* = 21.5 Hz), 62.5 (d, *J* = 6.7 Hz), 33.6, 31.7, 25.2, 18.0, 16.0 (d, *J* = 5.9 Hz), -5.1; **^{19}F NMR** (282 MHz, $CDCl_3$) δ_F -137.4 (dd, *J* = 10.7, 8.2 Hz); **m/z** HRMS (ESI) found [M+H]+, 434.1390, $C_{18}H_{30}NFO_4PSSi$ requires 434.1386

Tert-butyl (5-((1_E_,3_E_)-4-(6-((tert-butyldimethylsilyl)oxy)-5-fluorobenzo[d]thiazol-2-yl)buta-1,3-dien-1-yl)pyridin-2-yl)carbamate

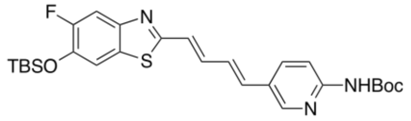

To a solution of diethyl ((6-((tert-butyldimethylsilyl)oxy)-5-fluorobenzo[d]thiazol-2-yl)methyl)phosphonate (174 mg, 0.402 mmol) in tetrahydrofuran (1.0 mL) at -78 ˚C was added lithium bis(trimethylsilyl)amide (1M solution in tetrahydrofuran, 420 µL). The solution was stirred at room temperature for 1 h after which the solution was cooled to -78 ˚C and a solution of (*E*)-*tert*-butyl (5-(3-oxoprop-1-en-1-yl)pyridin-2-yl)carbamate (66 mg, 0.268 mmol) in tetrahydrofuran (1.5 mL) was added. The solution was warmed to room temperature and stirred for 16 h. Acetic acid was then added until the solution reached pH 7 followed by addition of water. The aqueous layer extracted with ethyl acetate and the combined organic layers were washed with saturated aqueous sodium bicarbonate, dried (Na_2SO_4) and concentrated *in vacuo*. The crude product was purified by flash column chromatography (10-20% ethyl acetate/petroleum ether) to afford the title compound (90 mg, 0.172 mmol, 64%). *Nb. TBS group can cleave in reaction therefore may be purified off and re-protected under standard conditions.*

^{1}H NMR (300 MHz, $CDCl_3$) δ_H 8.27 (1H, d, *J* = 2.2 Hz), 8.03 (1H, d, *J* = 8.8 Hz), 7.87 (1H, d, *J* = 9.3 Hz), 7.80 (1H, br s), 7.64 (1H, d, *J* = 11.0 Hz), 7.20 – 7.33 (2H, m), 6.88 – 6.98 (2H, m), 6.77 (1H, d, *J* = 15.9 Hz), 1.54 (9H, s), 1.03 (9H, s), 0.23 (6H, s); **m/z** HRMS (ESI) found [M+H]+, 528.2135, $C_{27}H_{34}FN_3O_3SSi$ requires 528.2147

5-((1_E_,3_E_)-4-(6-((_tert_-butyldimethylsilyl)oxy)-5-fluorobenzo[d]thiazol-2-yl)buta-1,3-dien-1-yl)pyridin-2-amine

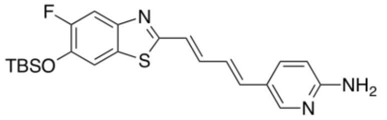

To a solution of *tert*-butyl (5-((1*E*,3*E*)-4-(6-((tert-butyldimethylsilyl)oxy)-5-fluorobenzo[d]thiazol-2-yl)buta-1,3-dien-1-yl)pyridin-2-yl)carbamate (60 mg, 0.114 mmol) in dichloromethane (1.2 mL) was added anisole (158 µL, 1.46 mmol) followed by trifluoroacetic acid (549 µL, 7.17 mmol) and the solution was stirred at room temperature for 16 h. Saturated aqueous sodium bicarbonate was added and the aqueous layer extracted with ethyl acetate. The combined organic layers dried (Na_2SO_4) and concentrated *in vacuo*. The crude product was purified by recrystallization from water-ethanol (5:1) to afford the title compound (35 mg, 0.0820, 72%).

^{1}H NMR (500 MHz, DMSO-d$_6$) δ_H 8.04 (1H, d, *J* = 2.2 Hz), 7.72 – 7.80 (2H, m), 7.68 (2H, dd, *J* = 8.7 2.2 Hz), 7.31 (1H, dd, *J* = 15.5, 8.7 Hz), 6.83 – 6.99 (3H, m), 6.47 (1H, d, *J* = 8.7 Hz), 6.37 (2H, s), 0.99 (9H, s), 0.22 (6H, s); **^{13}C NMR** (125 MHz, DMSO-d$_6$) δ_C 160.3, 150.1 (d, *J* = 215 Hz), 139.2, 138.5, 136.3, 135.7, 134.6, 130.4 123.7, 123.1, 121.3, 114.2, 109.4 (d, *J* = 10.6 Hz), 109.2 (d, *J* = 10.0 Hz), 109.0 (d, *J* = 3.0 Hz), 108.0 (d, *J* = 1.8 Hz), 26.1, 18.4, 2.7; **^{19}F NMR** (282 MHz, DMSO-d$_6$) δ_F -132.5 (dd, *J* = 11.1, 9.1 Hz); **m/z** HRMS (ESI) found [M+H]+, 428.1616, $C_{22}H_{27}FN_3OSSi$ requires 428.1623

5-((1*E*,3*E*)-4-(6-((*tert*-butyldimethylsilyl)oxy)-5-fluorobenzo[d]thiazol-2-yl)buta-1,3-dien-1-yl)-N-methylpyridin-2-amine

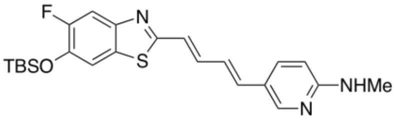

To a solution of diethyl ((6-((*tert*-butyldimethylsilyl)oxy)-5-fluorobenzo[d]thiazol-2-yl)methyl)phosphonate (100 mg, 0.231 mmol) in tetrahydrofuran (1.0 mL) at -78 ˚C was added lithium bis(trimethylsilyl)amide (1M solution in tetrahydrofuran, 262 µL). The solution was stirred at room temperature for 1 h after which the solution was cooled to -78 ˚C and a solution of (*E*)-3-(6-(methylamino)pyridin-3-yl)acrylaldehyde (31 mg, 0.193 mmol) in tetrahydrofuran (1.0 mL) was added. The solution was warmed to room temperature and stirred for 16 h. Acetic acid was then added until the solution reached pH 7 followed by addition of water. The aqueous layer extracted with ethyl acetate and the combined organic layers were washed with saturated aqueous sodium bicarbonate, dried (Na_2SO_4) and concentrated *in vacuo*. The crude product was used directly in the next step without purification.

^{1}H NMR (300 MHz, DMSO-d$_6$) δ_H 8.10 (1H, d, *J* = 2.5 Hz), 7.68 – 7.79 (2H, m), 7.51 (1H, d, *J* = 8.5 Hz), 7.21 – 7.36 (1H, m), 6.81 – 7.00 (4H, m), 6.49 (1H, d, *J* = 8.9 Hz), 2.80 (3H, d, *J* = 5.3 Hz), 0.99 (9H, s), 0.22 (6H, s).

LM3-229 (LM229)

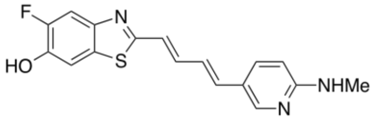

Prepared according to General Procedure B using 5-((1*E*,3*E*)-4-(6-((*tert*-butyldimethylsilyl)oxy)-5-fluorobenzo[d]thiazol-2-yl)buta-1,3-dien-1-yl)-N-methylpyridin-2-amine (50 mg, 0.113 mmol), pyridine (110 µL), HF-pyridine (110 µL) and tetrahydrofuran (240 µL). Recrystallization from water-ethanol afforded the title compound (25 mg, 0.0765, 68%).

^{1}H NMR (500 MHz, DMSO-d$_6$) δ_H 10.3 (1H, s), 8.10 (1H, d, *J* = 2.3 Hz), 7.71 (1H, d, *J* = 11.6 Hz), 7.70 (1H, dd, *J* = 8.6, 2.3 Hz), 7.52 (1H, d, *J* = 8.5 Hz), 7.7 (1H, dd, *J* = 15.4, 9.4 Hz), 6.82 – 6.99 (4H, m), 6.49 (1H, d, *J* = 8.6 Hz), 2.81 (3H, d, *J* = 4.8 Hz); **^{13}C NMR** (125 MHz, DMSO-d$_6$) δ_C 165.8, 159.8, 151.9 (d, *J* = 241. 7 Hz), 149.1, 146.9 (d, *J* = 9.8 Hz), 144.6 (d, *J* = 14.1 Hz), 138.5, 135.8, 134.0, 130.4 (d, *J* = 1.4 Hz), 123. 7, 123.1, 120.9, 109.3 (d, *J* = 21.1 Hz), 109.0 (d, *J* = 3.4), 108.7, 28.3; **^{19}F NMR** (282 MHz, DMSO-d$_6$) δ_F -135.8 (dd, *J* = 11.6, 8.5 Hz); **m/z** HRMS (ESI) found [M+H]+, 328.0916, $C_{17}H_{15}ON_3FS$ requires 328.0914

5-((1E,3E)-4-(5-fluorobenzo[d]thiazol-2-yl)buta-1,3-dien-1-yl)-N-methylpyridin-2-amine (LM2-141)

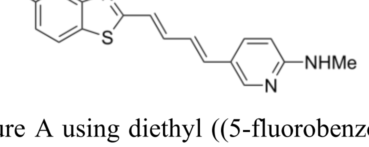

Prepared according to General Procedure A using diethyl ((5-fluorobenzothiazol-2-yl)methyl)phosphonate (112 mg, 0.369 mmol) and lithium bis(trimethylsilyl)amide (355 µL) in tetrahydrofuran (400 µL), (*E*)-3-(6-(methylamino)pyridin-3-yl)acrylaldehyde (40 mg, 0.246 mmol), and tetrahydrofuran (1.2 mL). Recrystallization from ethyl acetate afforded the title compound (30 mg, 0.0965 mmol, 39%).

^{1}H NMR (300 MHz, DMSO-d$_6$) δ_H 8.12 (1H, d, *J* = 2.1 Hz), 8.08 (1H, dd, *J* = 9.5, 5.0 Hz), 7.69 – 7.77 (2H, m), 7.42 (1H, ddd, *J* = 15.3, 6.4, 3.5 Hz), 7.29 (1H, td, *J* = 9.2, 2.6 Hz), 6.87 – 7.02 (4H, m), 6.49 (1H, d, *J* = 8.9 Hz), 2.81 (3H, d, *J* = 4.9 Hz); **^{13}C NMR** (75 MHz, DMSO-d$_6$) δ_C 169.5, 161.3 (d, *J* = 241.1 Hz), 159.4, 154.6 (d, *J* = 12.9 Hz), 148.9, 139.9, 136.6, 133.6, 129.6 (d, *J* = 1.7 Hz), 123.4, 123.2, 123.0, 122.2, 120.3, 113.4 (d, *J* = 25.1 Hz), 108.2 (d, *J* = 25.1 Hz); **^{19}F NMR** (282 MHz, DMSO-d$_6$) δ_F -116.1 (td, *J* = 9.5, 5.5 Hz); **m/z** HRMS (ESI) found [M+H]+, 312.0967, $C_{17}H_{14}N_3FS$ requires 312.0965

2-((1E,3E)-4-(6-fluoropyridin-3-yl)buta-1,3-dien-1-yl)benzo[d]thiazol-6-ol (LM2-148)

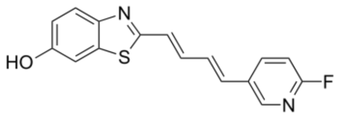

Prepared according to General Procedure B using 6-((*tert*-butyldimethylsilyl)oxy)-2-((1*E*,3*E*)-4-(6-fluoropyridin-3-yl)buta-1,3-dien-1-yl)benzothiazole (40 mg, 0.0971 mmol), pyridine (94 µL), HF-pyridine (94 µL) and tetrahydrofuran (200 µL). Recrystallization from water-ethanol afforded the title compound (25 mg, 0.0839 mmol, 86%).

^{1}H NMR (300 MHz, DMSO-d$_6$) δ_H 9.92 (1H, br s), 8.38 (1H, d, *J* = 2.6 Hz), 8.24 (1H, td, *J* = 8.0, 2.2 Hz), 7.76 (1H, d, *J* = 8.6 Hz), 7.36 (1H, d, *J* = 2.6 Hz), 7.22 – 7.33 (3H, m), 7.00 – 7.08 (2H, m), 6.96 (1H, dd, *J* = 8.6, 2.6 Hz); **^{13}C NMR** (75 MHz, DMSO-d$_6$) δ_C 164.1 (d, *J* = 200.0 Hz), 156.5, 147.5, 146.8 (d, *J* = 14.5 Hz), 139.5 (d, *J* = 7.8 Hz), 136.7, 136.1, 135.4 (d, *J* = 1.1 Hz), 131.9, 131.5 (d, *J* = 4.6 Hz), 130.2, 127.0, 123.8, 116. 6, 110.5 (d, *J* = 38.0 Hz), 107.1; **^{19}F NMR** (282 MHz, DMSO-d$_6$) δ_F -69.17 (dd, *J* = 8.0, 2.6 Hz); **m/z** HRMS (ESI) found [M+H]+, 299.0646, $C_{16}H_{12}N_2FOS$ requires 299.0649.

2-((1E,3E)-4-(5-fluoropyridin-2-yl)buta-1,3-dien-1-yl)benzo[d]thiazol-6-ol (LM2-152)

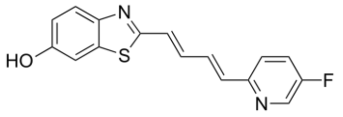

Prepared according to General Procedure B using 6-((*tert*-butyldimethylsilyl)oxy)-2-((1*E*,3*E*)-4-(5-fluoropyridin-2-yl)buta-1,3-dien-1-yl)benzothiazole (30 mg, 0.0728 mmol), pyridine (71 µL), HF-pyridine (71 µL) and tetrahydrofuran (150 µL). Recrystallization from water-ethanol afforded the title compound (10 mg, 0.0336 mmol, 46%).

^{1}H NMR (300 MHz, DMSO-d$_6$) δ_H 9.94 (1H, br s), 8.59 (H, d, *J* = 3.1 Hz), 7.77 (1H, d, *J* = 8.8 Hz), 7.75 (1H, td, *J* = 8.4, 2.8 Hz), 7.63 (1H, dd, *J* = 8.9, 4.8 Hz), 7.49 (1H, dd, *J* = 15.0, 10.7 Hz), 7.37 (1H, d, *J* = 2.3 Hz), 7.33 (1H, dd, *J* = 15.2, 10.9 Hz), 7.14 (1H, d, *J* = 15.0 Hz), 7.08 (1H, d, *J* = 15.2 Hz), 6.97 (1H, dd, *J* = 8.62.3 Hz); **^{13}C NMR** (75 MHz, DMSO-d$_6$) δ_C 162.8, 156.5, 147.5, 138.4 (d, *J* = 24.5 Hz), 136.2, 136.1 (d, *J* = 13.9 Hz), 136.0, 135.0, 131.8, 131.6, 128.2, 124.5, 124.1, 123.8, 116.6, 107.1; **^{19}F NMR** (282 MHz, DMSO-d$_6$) δ_F -127.8 (dd, *J* = 8.8, 4.8 Hz); **m/z** HRMS (ESI) found [M+H]+, 299.0649, $C_{16}H_{12}N_2FOS$ requires 299.0649.

2-((1E,3E)-4-(5-fluoropyridin-2-yl)buta-1,3-dien-1-yl)benzo[d]thiazole (LM2-164)

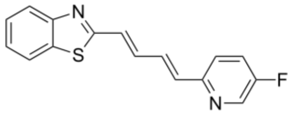

Prepared according to General Procedure A using diethyl (benzothiazol-2-ylmethyl)phosphonate (62 mg, 0.249 mmol) and lithium bis(trimethylsilyl)amide (244 µL) in tetrahydrofuran (300 µL), and (*E*)-3-(5-fluoropyridin-2-yl)acrylaldehyde (25 mg, 0.166 mmol) in tetrahydrofuran (2.0 mL). Recrystallization from water-ethanol afforded the title compound (15 mg, 0.0532 mmol, 32%).

^{1}H NMR (300 MHz, DMSO-d$_6$) δ_H 8.60 (1H, d, *J* = 3.4 Hz), 8.10 (1H, ddd, *J* = 8.0, 1.3, 0.6 Hz), 7.98 (1H, ddd, *J* = 8.0, 1.4, 0.6 Hz), 7.77 (1H, td, *J* = 8.8, 3.0 Hz), 7.65 (1H, dd, *J* = 8.7, 4.7 Hz), 7.42 – 7.58 (4H, m), 7.10 – 7.30 (2H, m); **^{13}C NMR** (75 MHz, DMSO-d$_6$) δ_C 166.5, 157.5, 155.7 (d, *J* = 241.5 Hz), 151.6, 138.5 (d, *J* = 24.6), 137.9, 136.0, 134.6, 131.5, 127.8 (d, *J* = 1.0 Hz), 127.1, 126.1, 124.7 (d, *J* = 4.6 Hz), 124.2 (d, *J* = 18.9 Hz), 123.1, 122.7; **^{19}F NMR** (282 MHz, DMSO-d$_6$) δ_F -127.5 (dd, *J* = 8.7, 4.8 Hz); **m/z** HRMS (ESI) found [M+H]+, 283.0699, $C_{16}H_{11}N_2FS$ requires 283.0700.

2-((1E,3E)-4-(5-fluoropyridin-2-yl)buta-1,3-dien-1-yl)benzo[d]thiazol-5-ol (LM2-166)

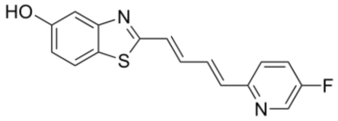

Prepared according to General Procedure B using 5-((*tert*-butyldimethylsilyl)oxy)-2-((1*E*,3*E*)-4-(5-fluoropyridin-2-yl)buta-1,3-dien-1-yl)benzothiazole (60 mg, 0.146 mmol), pyridine (142 µL), HF-pyridine (142 µL) and tetrahydrofuran (300 µL). Recrystallization from water-ethanol afforded the title compound (22 mg, 0.0738 mmol, 51%).

^{1}H NMR (300 MHz, DMSO-d$_6$) δ_H 9.72 (1H, s), 8.59 (1H, d, *J* = 2.9 Hz), 7.83 (1H, d, *J* = 8.5 Hz), 7.75 (1H, td, *J* = 8.8, 3.0 Hz), 7.63 (1H, dd, *J* = 8.5, 4.7 Hz), 7.39 – 7.54 (2H, m), 7.29 (1H, d, *J* = 2.5 Hz), 7.08 – 7.19 (2H, m), 6.93 (1H, dd, *J* = 8.8, 2.5 Hz); **^{13}C NMR** (75 MHz, DMSO-d$_6$) δ_C 167.1, 158.8 (d, *J* = 254.2 Hz), 157.2, 155.5, 151.6 (d, *J* = 4.4 Hz), 138.5 (d, *J* = 24.0 Hz), 137.1, 135.7, 131.6 (d, *J* = 2.3 Hz), 128.0, 124. 8, 124.6 (d, *J* = 4.5 Hz), 124.3 (d, *J* = 18.5 Hz), 122.8, 116.1, 108.0; **^{19}F NMR** (282 MHz, DMSO-d$_6$) δ_F -127.7 (dd, *J* = 8.7, 4.8 Hz); **m/z** HRMS (ESI) found [M+H]+, 299.0650, $C_{16}H_{12}N_2FOS$ requires 299.0649.

2-((1E,3E)-4-(6-fluoropyridin-3-yl)buta-1,3-dien-1-yl)benzo[d]thiazole (LM2-169)

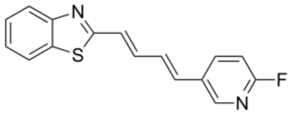

Prepared according to General Procedure A using (benzothiazol-2-ylmethyl)phosphonate (25 mg, 0.100 mmol) and lithium bis(trimethylsilyl)amide (98 µL) in tetrahydrofuran (150 µL), and (*E*)-3-(6-fluoropyridin-3-yl)acrylaldehyde (10 mg, 0.0662) in tetrahydrofuran (1.0 mL). Recrystallization from water-ethanol afforded the title compound (5 mg, 0.0177 mmol, 27%).

^{1}H NMR (300 MHz, DMSO-d$_6$) δ_H 8.41 (1H, d, *J* = 2.4 Hz), 8.26 (1H, td, *J* = 8.2, 2.5 Hz), 8.09 (1H, ddd, *J* = 7.7, 1.2, 0.6 Hz), 7.97 (1H, ddd, *J* = 8.2, 1.3, 0.6 Hz), 7.30 – 7.54 (4H, m) 7.25 (1H, dd, *J* = 8.7, 3.0 Hz), 7.10 (2H, d, *J* = 15.0 Hz); **^{13}C NMR** (75 MHz, DMSO-d$_6$) δ_C 168.1, 155.9 (d, *J* = 239.0 Hz), 146.7, 139.5 (d, *J* = 30.6 Hz), 137.5, 136.8, 133.0, 130.9, 130.1, 127.1, 124.4 (d, *J* = 24.0 Hz), 123.1, 122.7, 121.5, 112.6, 11.9; **^{19}F NMR**

(282 MHz, DMSO-d$_6$) δ_F -68.9 (dd, *J* = 8.0, 2.6 Hz); **m/z** HRMS (ESI) found [M+H]+, 283.0701, $C_{16}H_{11}N_2FS$ requires 283.0700.

5-((1*E*, 3*E*)-4-(6-fluorobenzothiazol-2-yl)buta-1,3-dien-1-yl)-*N*-methylpyridin-2-amine (LM3-207)

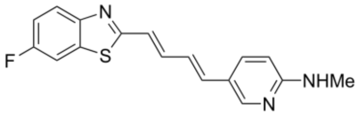

Prepared according to General Procedure A using diethyl ((6-fluorobenzothiazol-2-yl)methyl)phosphonate (134 mg, 0.442 mmol) and lithium bis(trimethylsilyl)amide (500 µL) in tetrahydrofuran (900 µL), and (*E*)-3-(6-(methylamino)pyridin-3-yl)acrylaldehyde (60 mg, 0.369) in tetrahydrofuran (3 mL). Recrystallization from water-ethanol afforded the title compound (42 mg, 0.135 mmol, 37%).

^{1}H NMR (300 MHz, DMSO-d$_6$) δ_H 8.11 (1H, d, *J* = 1.7 Hz), 7.97 (1H, dd, *J* = 8.9, 2.7 Hz), 7.92 (1H, dd, *J* = 9.0, 5.0 Hz), 7.70 (1H, dd, *J* = 9.0, 2.4 Hz), 7.31 – 7.41 (2H, m), 6.86 – 7.01 (4H, m), 6.49 (1H, d, *J* = 8.9 Hz), 2.81 (3H, d, *J* = 5.0 Hz); **^{13}C NMR** (75 MHz, DMSO-d$_6$) δ_C 167.3 (d, *J* = 3.7 Hz), 160.1 (d, *J* = 242.7 Hz), 159.8, 150.9 (d, *J* = 1.6 Hz), 149.3, 139.9, 136.6, 135.6 (d, *J* = 11.8 Hz), 134.0, 123.8 (d, *J* = 9.4 Hz), 123.5, 122.7, 120.8, 115.3 (d, *J* = 23.6 Hz), 109.1, 108.8, 28.3; **^{19}F NMR** (282 MHz, DMSO-d$_6$) δ_F -116.2 (td, *J* = 9.0, 4.9 Hz); **m/z** HRMS (ESI) found [M+H]+, 312.0962, $C_{17}H_{15}N_3FS$ requires 312.0965.

5-((1*E*,3*E*)-4-(5-fluoro-6-methoxybenzothiazol-2-yl)buta-1,3-dien-1-yl)-*N*-methylpyridin-2-amine (LM3-225)

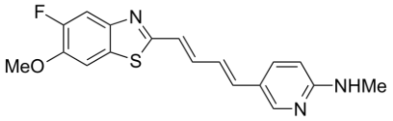

Prepared according to General Procedure A using diethyl ((5-fluoro-6-methoxybenzothiazol-2-yl)methyl)phosphonate (200 mg, 0.601 mmol) and lithium bis(trimethylsilyl)amide (683 µL) in tetrahydrofuran (1.2 mL), and (*E*)-3-(6-(methylamino)pyridin-3-yl)acrylaldehyde (81 mg, 0.502 mmol) in tetrahydrofuran (4 mL). Flash column chromatography (50% ethyl acetate/hexane) afforded the title compound (92 mg, 0.270 mmol, 54%).

^{1}H NMR (500 MHz, DMSO-d$_6$) δ_H 8.10 (1H, d, *J* = 2.0 Hz), 7.87 (1H, d, *J* = 8.4 Hz), 7.78 (1H, d, *J* = 11.9 Hz), 7.70 (1H, dd, *J* = 8.9, 2.5 Hz), 7.31 (1H, ddd, *J* = 15.4, 8.1, 1.4 Hz), 6.83 – 7.00 (4H, m), 6.49 (1H, d, *J* = 8.8 Hz), 3.91 (3H, s), 2.80 (3H, d, *J* = 4.8 Hz); **^{13}C NMR** (125 MHz, DMSO-d$_6$) δ_C 166.8, 159.9, 152.0 (d, *J* = 245.8 Hz), 149.2, 147.5 (d, *J* = 10.3 Hz), 146.6 (d, *J* = 11.7), 139.0, 136.0, 136.2, 134.1, 130.5, 123.6, 122.9, 121.0, 109.1 (d, *J* = 20.7 Hz), 108.8, 106.1, 56.9, 28.2; **^{19}F NMR** (282 MHz, DMSO-d$_6$) δ_F -135.3 (dd, *J* = 11.8, 8.5 Hz); **m/z** HRMS (ESI) found [M+H]+, 342.1061, $C_{18}H_{16}N_3FOS$ requires 342.1071.

5-((1*E*, 3*E*)-4-(5-(2-fluoroethoxy)benzothiazol-2-yl)buta-1,3-dien-1-yl)-*N*-methylpyridin-2-amine (LM3-236)

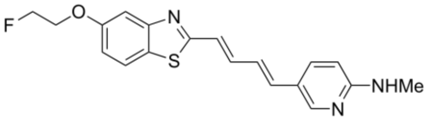

Prepared according to General Procedure A using diethyl ((5-(2-fluoroethoxy)benzothiazol-2-yl)methyl)phosphonate (250 mg, 0.720 mmol) and lithium bis(trimethylsilyl)amide (818 µL) in tetrahydrofuran

(1.4 mL), and (*E*)-3-(6-(methylamino)pyridin-3-yl)acrylaldehyde (97 mg, 0.601) in tetrahydrofuran (5 mL). Recrystallization from water-ethanol afforded the title compound (73 mg, 0.206 mmol, 34%).

H NMR (300 MHz, DMSO-d$_6$) δ_H 8.11 (1H, d, *J* = 1.9 Hz), 7.92 (1H, d, *J* = 8.5 Hz), 7.71 (1H, d, *J* = 8.9 Hz), 7.50 (1H, d, *J* = 1.9 Hz), 7.37 (1H, dd, *J* = 15.1, 8.9 Hz), 7.07 (1H, dd, *J* = 8.9, 2.4 Hz), 6.86 – 7.01 (4H, m), 6.49 (1H, d, *J* = 8.9 Hz), 4.79 (2H, dt, *J* = 47.7, 3.6), 4.33 (2H, dt, *J* = 30.3, 3.6 Hz), 2.81 (3H, d, *J* = 4.8 Hz); ^{13}**C NMR** (75 MHz, DMSO-d$_6$) δ_C 168.4, 159.8, 158.0, 155.4, 149.2, 139.4, 136.4, 134.0, 126.4, 123.6, 123.0, 122.9, 120.9, 115.5, 108.7, 106.4, 82.6 (d, *J* = 165.2 Hz), 68.0 (d, *J* = 18.7 Hz), 28.3; ^{19}**F NMR** (282 MHz, DMSO-d$_6$) δ_F -221.9 (tt, *J* = 48.1, 30.6 Hz); **m/z** HRMS (ESI) found [M+H]+, 356.1218, $C_{19}H_{19}N_3FOS$ requires 356.1227.

2-((1*E*,3*E*)-4-(6-(methylamino)pyridin-3-yl)buta-1,3-dien-1-yl)benzothiazol-5-ol (LM2-146)

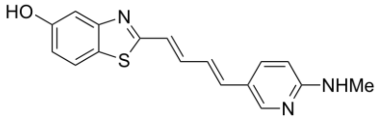

Prepared according to General Procedure B using 5-((1*E*,3*E*)-4-(5-((*tert*-butyldimethylsilyl)oxy)benzothiazol-2-yl)buta-1,3-dien-1-yl)-*N*-methylpyridin-2-amine (30 mg, 0.0709 mmol), pyridine (69 µL), HF-pyridine (69 µL) and tetrahydrofuran (150 µL). Recrystallization from water-ethanol afforded the title compound (15 mg, 0.0485 mmol, 68%).

^{1}**H NMR** (300 MHz, DMSO-d$_6$) δ_H 9.68 (1H, s), 8.10 (1H, d, *J* = 2.0 Hz), 7.78 (1H, d, *J* = 8.8 Hz), 7.70 (1H, dd, *J* = 8.8, 2.4 Hz), 7.33 (1H, dd, *J* = 15.6, 8.8 Hz), 7.23 (1H, d, *J* = 2.4 Hz), 6.82 – 6.99 (5H, m), 6.49 (1H, d, *J* = 9.2 Hz), 2.80 (3H, d, *J* = 4.5 Hz); ^{13}**C NMR** (75 MHz, DMSO-d$_6$) δ_C 167.9, 159.8, 157.1, 155.6, 149.2, 139.1, 136.2, 134.0, 124.5, 123.6, 123.2, 122.6, 120.9, 115.6, 108.8, 107.8, 28.4; **m/z** HRMS (ESI) found [M+H]+, 310.1009, $C_{17}H_{16}N_3OS$ requires 310.1009.

2-((1*E*,3*E*)-4-(pyridin-3-yl)buta-1,3-dien-1-yl)benzothiazol-6-ol (LM2-165)

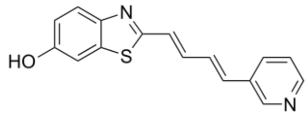

Prepared according to General Procedure B using 6-((*tert*-butyldimethylsilyl)oxy)-2-((1*E*,3*E*)-4-(pyridin-3-yl)buta-1,3-dien-1-yl)benzothiazole (34 mg, 0.0863 mmol), pyridine (84 µL), HF-pyridine (84 µL) and tetrahydrofuran (180 µL). Recrystallization from water-ethanol afforded the title compound (16 mg, 0.0571 mmol, 66%).

^{1}**H NMR** (300 MHz, DMSO-d$_6$) δ_H 9.94 (1H, br s), 8.73 (1H, d, *J* = 1.5 Hz), 8.48 (1H, dd, *J* = 5.9, 1.5 Hz), 8.01 (1H, d, *J* = 8.3 Hz), 7.76 (1H, d, *J* = 8.8 Hz), 7.42 (1H, dd, *J* = 8.3, 5.4 Hz), (7.25 – 7.36 (3H, m), 7.00 – 7.09 (2H, m), 6.96 (1H, dd, *J* = 8.3, 2.0 Hz); ^{13}**C NMR** (75 MHz, DMSO-d$_6$) δ_C 163.3, 162.8, 156.5, 149.5, 149.0, 147.6, 136.8, 136.1, 133.5, 133.5, 130.4, 127.1, 124.4, 123.8, 116.6, 107.2; **m/z** HRMS (ESI) found [M+H]+, 281.0744, $C_{16}H_{13}N_2OS$ requires 281.0743.

5-((1*E*,3*E*)-4-(6-methoxybenzothiazol-2-yl)buta-1,3-dien-1-yl)-*N*-methylpyridin-2-amine (LM2-186)

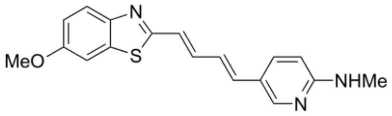

Prepared according to General Procedure A using diethyl ((6-methoxybenzothiazol-2-yl)methyl)phosphonate (64 mg, 0.231 mmol) and lithium bis(trimethylsilyl)amide (333 µL) in tetrahydrofuran (500 µL), and (*E*)-3-(6-(methylamino)pyridin-3-yl)acrylaldehyde (25 mg, 0.154 mmol) in tetrahydrofuran (1.8 mL). Recrystallization from water-ethanol afforded the title compound (28 mg, 0.0867 mmol, 56%).

^{1}H NMR (300 MHz, DMSO-d$_6$) δ_H 8.10 (1H, d, *J* = 1.3 Hz), 7.79 (1H, d, *J* = 8.6 Hz), 7.70 (1H, dd, *J* = 8.6, 1.7 Hz), 7.63 (1H, d, *J* = 2.2 Hz), 7.28 (1H, dd, *J* = 15.5, 9.5 Hz), 7.07 (1H, dd, *J* = 8.6, 2.2 Hz), 6.83 – 6.99 (4H, m), 6.49 (1H, d, *J* = 9.0 Hz), 3.83 (3H, s), 2.80 (3H, d, *J* = 4.7 Hz); **^{13}C NMR** (75 MHz, DMSO-d$_6$) δ_C 164.7, 159.8, 157.8, 149.1, 148.5, 138.7, 135.8, 136.8, 134.0,123.7,123.3, 123.2, 120.9, 116.0, 108.8, 105.3, 56.2, 28.4; **m/z** HRMS (ESI) found [M+H]+, 324.1174, $C_{18}H_{18}N_3OS$ requires 324.1165.

HPLC Radio-chromatogram Analysis of purified [^{11}C]LM229 product

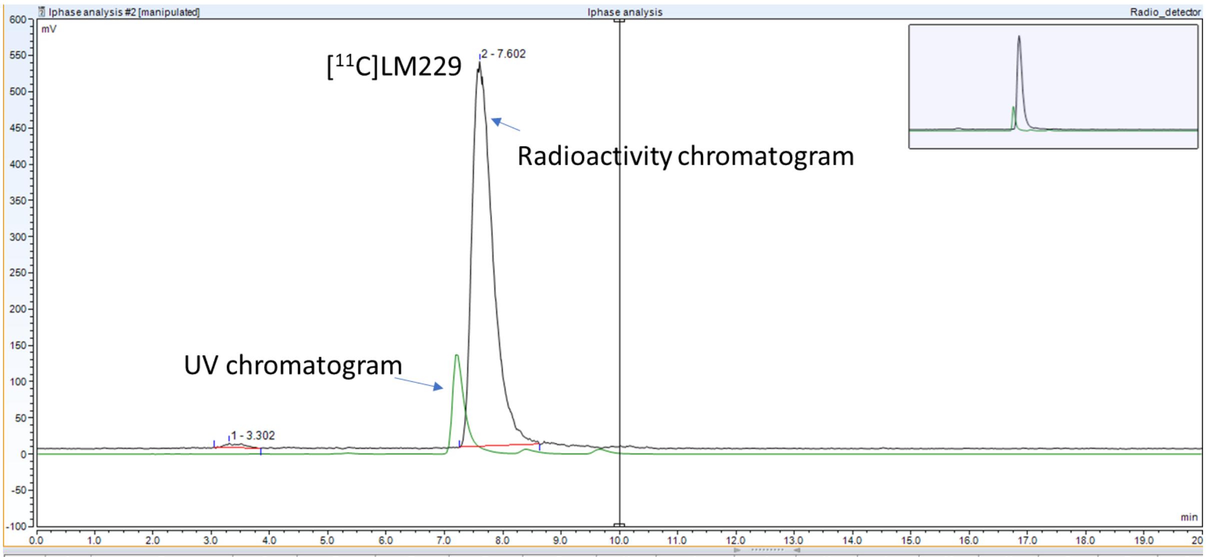

Peak No.	Peak Name	Ret.Time min	Amount n.a.	Rel.Area %	Area mV*min	Height mV	Type	Width (50%) min	Asym. EP	Resol. EP	Plates EP
1		3.302	n.a.	0.97	2.1558	6.35	BMB*	0.382	2.00	6.72	415
2		7.602	n.a.	99.03	219.2628	531.31	BMB*	0.374	1.67	n.a.	2290
Maximum			0.0000	99.03	219.2628	531.31		0.382	2.00	6.72	2290
Minimum			0.0000	0.97	2.1558	6.35		0.374	1.67	6.72	415
Sum			0.0000	100.00	221.4187	537.67					

Figure S1. HPLC analysis of purified [^{11}C]LM229 with radioactivity detection and UV. Chromatograms superimposed.

Immunohistochemistry on P301CR mice

Brains were cut into 20 µM thick sections using a cryostat and collected directly onto slides. Tissue sections were blocked in PBS/0.1% Triton X-100 buffer containing 5% bovine serum albumin (blocking buffer) for 60 min. Slices were washed twice in PBS/0.1% Triton X-100. Tissues were then incubated with AT8 (1:1000 diluted in blocking buffer) overnight at 4 ˚C. Tissues were then washed in PBS/0.1% Triton X-100, followed by incubation with secondary antibody (Alexa Fluor555 conjugated anti-mouse IgG, diluted 1:1000 in blocking

buffer) for 1 h at room temperature. Following 3 washes in PBS/0.1% Triton X-100, 50 µM LM229 (diluted in 1:1 blocking buffer/ethanol) was then added for 1 h at room temperature. Nuclei were stained with DAPI (0.1mg/ml for 10 min), washed and tissues mounted in FluorSave^{TM} prior to analysis using fluorescence microscopy.

Binding affinity of all compounds with tau fibrils

Microscale Thermophoresis was first used to screen whether there were interaction between the compounds and tau fibrils. For this procedure 200 µL, 5 µM tau fibrils were mixed with 200 µL, 10 µM NT647-NHS dye and then incubated in dark for 30 minutes. Further dialysis (18 hours, changing the buffer after the first 4 hours) was used to remove the free dye giving a 2.5 µM solution of labelled protein. The labelling ratio of dye unit per tau monomer was then tested by a method recommended by the instrument manufacturer, NanoTemper Technologies, which is based on a calibration curve. The 2.5 µM tau fibril solution was then aliquoted prior to use. The unused samples were frozen at -20 °C. MST measurements were conducted using Monolith NT.115 instrument (NanoTemper Technologies, Germany) at 25 °C. Assays were conducted at 40 % MST Power and 90 % IR-laser power. With each compound, two concentrations (1µM and 1nM) were used to mix with 100nM tau fibrils and then loaded to the MST instrument to read out the MST data curve. Only three compounds 166, 164 and 169 showed no changes with the MST data curve. All other compounds binding affinities towards tau fibrils were then tested by fluorescent binding assay.

Binding to cultured neurons from P301S tau transgenic mice

DRG were incubated with the indicated concentrations of LM229 or PBB3 for 20 minutes at room temperature, washed 3 times in PBS, and imaged on a Leica DMI 4000B microscope using a Leica DFC3000 G camera and the Leica application suite 4.0.0.11706. Fluorescence intensity was measured in 10-16 neurons each from 3 independent cultures using Image J (imagej.nih.gov/ij/). Results shown are mean ± SD. Non linear least squares curve fitting was applied using http://faculty.gvsu.edu/carlsont/232lab/nonlin2.html. No Km value was determined for PBB3.

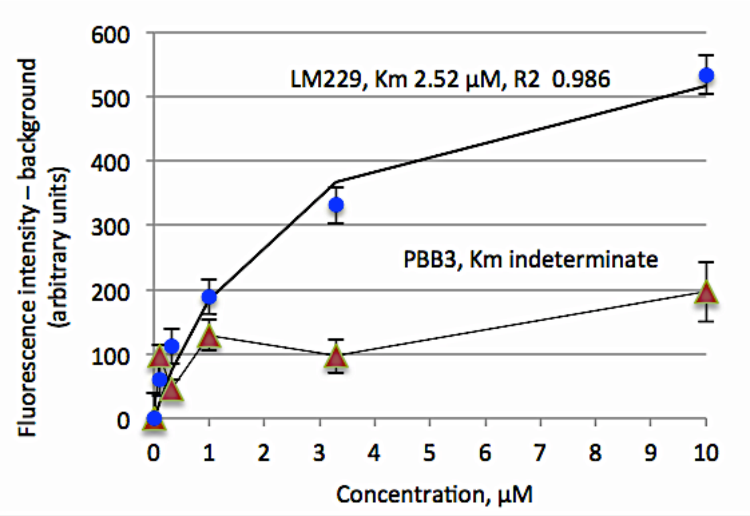

Figure S2: Binding of LM229 and PBB3 to DRG neurons with tau aggregates

Figure S3: Brainstem region of 2 MO (A) or 6 MO (B) P301S Tau tissue section stained with LM229 (left panels) or with AT100 (right panels) and counter-stained with DAPI. Scalebar = 100 μm.

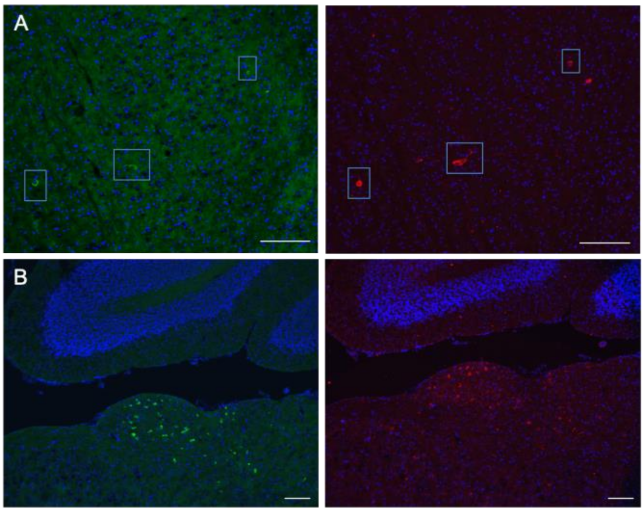

Supplement: Supplementary file 1 — cn0c00790_si_001.pdf [file cn0c00790_si_001.pdf]
